# Genetic variants of *CD160*, *MERTK*, and *IL15* in natural killer cell-related pathway predict gastric cancer survival

**DOI:** 10.3389/fimmu.2026.1765825

**Published:** 2026-04-15

**Authors:** Guang Zeng, Guoqiang Lu, Beiping Hu, Midie Xu, Guanlin Li, Mengyun Wang, Lixin Qiu, Ruoxin Zhang, Lei Cheng, Wanghong Xu, Xiaowen Liu, Guangfu Jin, Hongliang Liu, Qingyi Wei

**Affiliations:** 1 Department of Epidemiology, School of Public Health, and The Research Institute for Cancer Control and Prevention, Greater Bay Area Institute of Precision Medicine (Guangzhou), Fudan University, Shanghai, China; 2 Department of Epidemiology, Center for Global Health, School of Public Health, Nanjing Medical University, Nanjing, China; 3 Department of Pathology, Fudan University Shanghai Cancer Center, Shanghai, China; 4 Department of Oncology, Shanghai Medical College, Fudan University, Shanghai, China; 5 Department of Gastric Surgery, Fudan University Shanghai Cancer Center, Shanghai, China; 6 Cancer Institute, Fudan University Shanghai Cancer Center, Shanghai, China; 7 Department of Gastrointestinal Medical Oncology, Fudan University Shanghai Cancer Center, Shanghai, China; 8 Department of Epidemiology, School of Public Health; Key Laboratory of Public Health Safety of Ministry of Education, Fudan University, Shanghai, China; 9 Department of Pulmonary, Shanghai Chest Hospital, Shanghai Jiao Tong University, Shanghai, China

**Keywords:** gastric cancer, natural killer cell, prognosis, single nucleotide polymorphism, tumor microenvironment

## Abstract

**Background:**

Natural killer (NK) cells play a pivotal role in anti-tumor immunity; however, the prognostic significance of genetic variants in NK cell-related genes in gastric cancer (GC) remains largely uncertain.

**Methods:**

We systematically evaluated 12,476 single-nucleotide polymorphisms (SNPs) in 151 NK cell-related genes for their associations with overall survival (OS) in 2,211 Chinese GC patients with pathological tumor-node-metastasis (TNM) stage I-III, who were enrolled in the Shanghai genome-wide association study (GWAS). Significant variants were further validated in an independent Jiangsu GWAS cohort comprising 1,049 GC patients with TNM stage I-III. The prognostic value of independent SNPs was evaluated. Bioinformatic annotations were performed through expression QTL, splicing QTL, histone modification QTL, and methylation QTL analyses, as well as transcription factor binding, differential expression, functional enrichment, and immune infiltration analyses.

**Results:**

Three independent SNPs (*CD160* rs9728526 A>G, *MERTK* rs114788905 G>A, and *IL15* rs140007893 T>A) were significantly associated with GC OS, with adjusted hazard ratios of 1.16 (95% CI = 1.05-1.29, *P* = 0.006), 0.89 (95% CI = 0.81-0.99, *P* = 0.033), and 0.77 (95% CI = 0.62-0.97, *P* = 0.028), respectively. A combined analysis demonstrated a dose-dependent association between the number of unfavorable genotypes and poorer OS. Incorporating these SNPs improved the C-index, time-dependent AUC, net reclassification improvement (NRI), and integrated discrimination improvement (IDI) for OS prediction. Functional annotation indicated that the variant alleles exerted tissue- and immune cell-specific regulatory effects on gene expression, splicing, and transcription factor binding. The expression levels of *CD160*, *MERTK*, and *IL15* were correlated with immune cell infiltration within the tumor microenvironment in GC.

**Conclusions:**

We identified three novel SNPs in NK cell-related genes that independently predict GC survival, providing potential prognostic biomarkers for risk stratification in GC, although further validation is warranted.

## Introduction

Gastric cancer (GC) is the fifth most prevalent cancer and the fourth leading cause of cancer-related deaths worldwide. East Asia, in particular, exhibits a markedly high incidence of GC (1). Notably, China accounts for approximately 37% of global GC cases, with an estimated 359,000 new diagnoses and 260,000 GC-related deaths in 2022 (2). Given the combined influence of genetic susceptibility, population aging, and prevalent modifiable risk factors such as smoking, physical inactivity, *Helicobacter pylori* infection, and inadequate fruit and vegetable consumption, the total number of GC cases is expected to rise in China (3, 4). Moreover, around 64% of Chinese GC patients are diagnosed at an advanced stage, thereby often missing the opportunity for curative surgery and consequently experiencing a poor prognosis (5). Despite advancements in health promotion, screening, diagnostic techniques, and treatment (e.g., novel targeted therapies and next-generation immunotherapies), the most recent five-year survival rate for GC in China remains only 35.2% (6), significantly lower than in other high-incidence East Asian countries such as South Korea (72.1%) (7) and Japan (80.1%) (8). Therefore, identifying promising survival-associated biomarkers is crucial for improving prognostic assessment and enabling precise interventions for Chinese GC patients.

The intricate and dynamic interactions between GC cells and various immune cells are hallmarks of the highly heterogeneous tumor microenvironment (TME), which fosters an immunosuppressive milieu that promotes GC cell proliferation, survival, invasion, and migration, ultimately leading to a poor prognosis (9). NK cells are major innate cytotoxic lymphocytes with anti-tumor activity and are primarily categorized into CD56bright and CD56dim NK cells based on the expression of the surface molecule CD56. They play a vital role in identifying and eliminating infected or tumor cells. Like B cells and T cells, NK cells are integral components of tumor-infiltrating lymphocytes in the GC TME (9). They enhance innate anti-tumor immunity and activate the adaptive immune response against cancer through multiple mechanisms, including the release of perforin and granzymes, death receptor-mediated apoptosis, antibody-dependent cell-mediated cytotoxicity, cytokine production (e.g., IFN-γ), and the recruitment of dendritic cells (DCs) and T cells to the TME (10). Human NK cells display tumor-type-specific heterogeneity and play a crucial role in inhibiting tumor initiation, metastasis, and progression in GC (11). Previous studies have revealed that in GC patients, NK cell apoptosis is elevated, while proliferation, infiltration, and cytotoxicity are reduced (12, 13). Moreover, high NK cell infiltration is correlated with better prognosis in GC patients (13, 14), although certain NK cell subsets (e.g., CD57+ NK cells and CD57+ NKG2A+ NK cells) are associated with poor survival (15). However, the roles of NK cell-related genes in the progression and prognosis of GC remain largely unexplored.

Since Klein et al. (16) first applied genome-wide association studies (GWAS) to identify genetic variations associated with the risk of age-related macular degeneration, GWAS over the past two decades have played a pivotal role in elucidating the genetic basis of complex diseases, including GC, and advancing precision medicine (17). To date, GWAS have identified numerous genetic susceptibility loci and single nucleotide polymorphisms (SNPs) associated with GC risk, such as rs59133000 in *PRKAA1* at 5p13.1 and rs4072037 in *MUC1* at 1q22 (18). Increasing evidence further suggests that SNPs in key genes and pathways play a critical role in GC prognosis by regulating gene expression and influencing biological processes (19). Additionally, survival-associated SNPs represent promising biomarkers because of their noninvasive nature, early prognostic value, and clinical translational potential for guiding personalized treatment in GC patients (20). Nonetheless, the roles of SNPs in NK cell-related genes in GC survival remain unclear. Therefore, in this study, we hypothesized that SNPs in NK cell-related genes are associated with survival in GC patients with pathological tumor-node-metastasis (TNM) stage I-III. We tested this hypothesis using GWAS genotyping and survival data from two large-scale independent patient cohorts: the Shanghai and Jiangsu cohorts, in a two-stage analysis framework.

## Materials and methods

### Study populations

In the two-stage analysis, the discovery GWAS dataset was derived from the Shanghai cohort, conducted at the Fudan University Shanghai Cancer Center (FUSCC). This cohort included 2,370 Han Chinese patients with GC, recruited between January 1, 2006, and December 31, 2014. Seventy-six GC patients with TNM stage IV and 83 GC patients with missing critical demographic, clinical, or follow-up data were excluded from the survival analysis. Genomic DNA from all participants was genotyped using Illumina chips, with further details provided elsewhere (20, 21). Ultimately, 2,211 GC patients with TNM stage I-III, nearly all of whom had undergone surgical resection, were included in the analysis.

The validation GWAS dataset was obtained from the Jiangsu cohort, established by medical institutions in Jiangsu Province between January 1, 2005, and December 31, 2017. This cohort comprised 1,049 GC patients with TNM stage I-III, all of whom had undergone surgical resection, as previously described (20). Blood samples were genotyped using either Illumina or Affymetrix chips (18, 20). All patients across the two cohorts were diagnosed with stomach adenocarcinoma (STAD) and had no serious infections or comorbidities that could affect overall survival (OS), such as other cancers, diabetes, heart failure, myocardial infarction, cerebral apoplexy, or other significant health conditions. The detailed characteristics of GC patients in the independent Shanghai and Jiangsu cohorts are presented in **Supplementary Table 1**.

### Gene selection, SNP imputation, and quality control

NK cell-related genes were retrieved from the Molecular Signature Database (https://www.gsea-msigdb.org/gsea/msigdb/human/search.jsp) using the keyword “natural killer cell” and selecting “Homo sapiens” as the source species (accessed on June 18, 2025) (22). As detailed in **Supplementary Table 2**, 151 genes were selected as candidates for further survival analyses. We performed imputation using data from the 1000 Genomes Project (Phase 3) as the reference panel, with IMPUTE software (version 2.3.1). We used PLINK 2.0 (https://www.cog-genomics.org/plink/) to extract SNPs within these candidate genes and their ±2 kb flanking regions from the Shanghai GWAS dataset. We performed quality control using PLINK 1.9, with criteria including an imputation information score ≥ 0.8, a minor allele frequency (MAF) ≥ 5%, an individual call rate ≥ 95%, and a Hardy-Weinberg equilibrium (HWE) *P*-value ≥ 1 × 10^-5^. Consequently, a total of 12,476 candidate SNPs were selected for subsequent analyses.

### Functional prediction

Five online bioinformatics tools were used to predict the potential functions of the identified SNPs, including RegulomeDB 2.2 (https://www.regulomedb.org/), HaploReg v4.2 (https://pubs.broadinstitute.org/mammals/haploreg/haploreg.php), 3DSNP (http://omic.tech/3dsnpv2/), SNPinfo Web Server (https://snpinfo.niehs.nih.gov/snpinfo/snpfunc.html), and Ensembl Variant Effect Predictor (https://grch37.ensembl.org/Tools/VEP). The UCSC Genome Browser (https://genome.ucsc.edu/index.html) was used for visualization. Additionally, allele-specific transcription factor binding was predicted using the JASPAR database (https://jaspar.elixir.no/) (23).

### Prognostic value evaluation

To investigate the prognostic value of the three independent SNPs, we initially generated time-dependent receiver operating characteristic (ROC) curves and calculated the area under the curve (AUC) for the prediction of 5-year and 10-year survival, using the “timeROC” package. We compared the AUC values between SNPs and other independent prognostic factors for GC identified from a stepwise Cox analysis. Furthermore, ROC curves and time-dependent AUCs were generated to develop a survival prognostic model, and the C-index, net reclassification improvement (NRI), and integrated discrimination improvement (IDI) were calculated using the “survival”, “timeROC”, “survcomp”, “nricens”, and “survIDINRI” packages (24, 25). These analyses assessed the predictive power of clinical and genetic variables for GC survival at different time points.

### Quantitative trait loci analyses

Initially, expression QTL (eQTL) analyses were conducted to explore the relationships between mRNA expression and the genotypes of the identified SNPs, using both bulk RNA sequencing (RNA-seq) and single-cell RNA-seq data. The bulk RNA-seq eQTL analysis incorporated three components: data from the Genotype-Tissue Expression (GTEx) project, which include 407 normal stomach tissues, 614 esophageal mucosa tissues, and 800 whole blood samples; data from the eQTLGen database, which contained 37 datasets with 31,684 whole blood samples; and genotyping and mRNA expression data from lymphoblastoid cell lines in the 1000 Genomes Project (1000G), which included 373 European individuals and 158 East Asian individuals (i.e., 76 CHB and 82 JPT) (26, 27). To uncover more precise genetic regulatory mechanisms of SNPs on gene expression, single-cell eQTL (sc-eQTL) analysis was conducted using data from the scGaTE platform (http://ccra.njmu.edu.cn/scgate/) (28), which provides cell-type-specific eQTL data across 19 gastric cell types (immune and non-immune cells) in the gastric mucosal tissues of 203 individuals from the Eastern Chinese population. Moreover, three additional sc-eQTL databases, including the FIVEx platform (https://fivex.sph.umich.edu/) (29), the scQTLbase database (http://bioinfo.szbl.ac.cn/scQTLbase/Home/) (30), and the Single-cell eQTL Hub (https://www.sqraolab.com/scqtl/) (31) were used to further investigate immune cell-specific gene expression regulation by independent SNPs.

Subsequently, we utilized the FIVEx platform to conduct splicing QTL (sQTL) analyses to explore the cis-regulatory effects of independent SNPs on alternative splicing of the genes under investigation across blood, stomach, and various immune cell types. Finally, to evaluate the effect of independent SNPs on transcription factor binding, DNA methylation, and histone modification, the QTLbase (http://www.mulinlab.org/qtlbase/index.html) database was used to perform transcription factor binding QTL (bQTL), histone modification QTL (hQTL), and methylation QTL (mQTL) analyses (32). The available and visualized results of these analyses were obtained from the QTLbase platform.

### Differential expression analyses and survival

We first performed differential expression analyses between GC tumor tissues and adjacent normal tissues using omics data from The Cancer Genome Atlas (TCGA) database, applying both paired and unpaired tests. Meanwhile, we utilized the UCSC Xena database (https://xena.ucsc.edu), which integrates data from TCGA tumor samples, TCGA normal samples, and GTEx normal samples, to conduct further differential expression analyses using unpaired tests (33). Additionally, using data from the TCGA database, we further explored the relationship between SNP-associated gene expression and the TNM stage in STAD. Furthermore, to explore the association between mRNA expression levels and GC OS, we utilized the online Kaplan-Meier Plotter tool (http://kmplot.com/analysis/) with the recommended JetSet best probe sets, specifically 207840_at for *CD160*, 206028_s_at for *MERTK*, and 205992_s_at for *IL15* (34).

### Enrichment analysis

To investigate whether *CD160*, *MERTK*, and *IL15* influence specific biological processes, we conducted Gene Ontology (GO) enrichment analysis using data from TCGA and the CAMOIP database (http://www.zjyy-oncology.com:20002/) in 373 STAD patients (35). Additionally, we used the CAMOIP database to perform Gene Set Enrichment Analysis (GSEA) to explore associations between the expression of *CD160*, *MERTK*, and *IL15* and potential biological pathways. The visualized results of these analyses were derived from the CAMOIP platform.

### Immune infiltration analysis

To explore the potential roles of *CD160*, *MERTK*, and *IL15* in the GC TME, we performed immune infiltration analyses using RNA-seq data and clinical information from 375 STAD patients in the TCGA repository. We initially applied the ESTIMATE algorithm to investigate the association between the expression of *CD160*, *MERTK*, and *IL15* and three scores: the stromal score, immune score, and ESTIMATE score (36). Subsequently, the immunophenotypic score (IPS) (37), which integrates major histocompatibility complex (MHC) molecules, effector cells, suppressor cells, and immune checkpoint molecules, was employed to evaluate the immune status of STAD samples from the TCGA database, using the CAMOIP database. Additionally, we used the single-sample gene set enrichment analysis (ssGSEA) algorithm (38, 39) to assess the correlations between the expression of *CD160*, *MERTK*, and *IL15* and the infiltration levels of 24 immune cell types in the GC TME, as well as to compare the enrichment scores of NK cells between the low- and high-expression groups of *CD160*, *MERTK*, and *IL15*, and those of DCs between the low- and high-expression groups of *MERTK*. Moreover, the TISIDB database (http://cis.hku.hk/TISIDB/index.php) was used to explore and visualize the correlations between gene expression and the abundance of NK cells. Lastly, the Microenvironment Cell Populations counter (MCPcounter) algorithm (40) was applied specifically to compare the absolute abundance of endothelial cells and fibroblasts between the low- and high-expression groups of *IL15* in GC, using the CAMOIP database.

### Statistical analyses

In the present study, OS was the primary endpoint, defined as the time from GC diagnosis to death or the most recent follow-up. The last follow-up dates for the Shanghai and Jiangsu cohorts were May 31, 2025, and July 30, 2019, respectively. Survival data for GC patients who were alive or lost to follow-up were treated as censored observations. Given that the mortality rate in most subgroups was below 50%, the restricted mean survival time (RMST), defined as the average survival time up to a fixed time point in a target population, was calculated instead of the median survival time to estimate the expected survival time of GC patients using the R package survRM2 (version 4.4.1) (41, 42). In the discovery stage, we assessed the associations between 12,476 candidate SNPs and GC OS using a single-locus multivariable Cox proportional hazards regression analysis under an additive genetic model, implemented in the R package GenABEL (version 3.6.1). The Cox model was adjusted for available covariates, including age at diagnosis, sex, smoking status, drinking status, TNM stage, chemotherapy, radiotherapy, and principal components (PCs) 7 and 8 (**Supplementary Table 3**). A detailed description of smoking and drinking status is available elsewhere (43). Because SNPs were imputed with a high degree of linkage disequilibrium (LD), the Bayesian False Discovery Probability (BFDP), defined as the cost of false discoveries and nondiscoveries, was applied with a threshold of 0.80 (i.e., assigning a false nondiscovery cost four times higher than a false discovery) to correct for multiple testing and to filter potential false positives, as recommended (44). Moreover, we employed a prior probability of 0.10 to detect variant genotypes or minor alleles associated with hazard ratios (HRs), with an upper bound of 3.0 and a *P*-value of < 0.05 (44).

In the validation stage, we replicated the significant SNPs (*P*_adj_ ≤ 0.05 and BFDP ≤ 0.80) identified from the discovery dataset in the Jiangsu cohort, using the same Cox model. The model was adjusted for age at diagnosis, sex, TNM stage, tumor location (non-cardia vs. cardia), chemotherapy or radiotherapy, and the top 10 PCs. Subsequently, we performed a multivariable stepwise Cox regression analysis based on the Akaike information criterion (AIC) to investigate the independent associations between identified SNPs and GC OS, adjusting for age at diagnosis, sex, smoking status, drinking status, TNM stage, chemotherapy, radiotherapy, PC7, and PC8. Additionally, we combined the results from both the Shanghai and Jiangsu cohorts through an inverse-variance weighted meta-analysis. Cochran’s Q-test and the heterogeneity statistic (*I* ^(2)^) were used to evaluate inter-study heterogeneity. If no heterogeneity was detected (Q-test *P*_het_ > 0.10 and *I*^2^ < 50%), we applied a fixed-effects model; otherwise, we used a random-effects model. Furthermore, we applied Haploview 4.2 software (https://www.broadinstitute.org/haploview/haploview) and the LocusZoom tool (http://locuszoom.sph.umich.edu/) to generate Manhattan and regional association plots for visualizing the independent SNPs, respectively.

Finally, we evaluated the associations between genotypes of independent SNPs and GC OS using additive, dominant, and recessive genetic models in the Shanghai cohort. The unfavorable genotypes were defined as having HR > 1 and *P* < 0.05 in genotype models and were combined into a number of unfavorable genotypes (NUGs) (45). We then evaluated the cumulative effects of NUGs on GC survival, and Kaplan-Meier (KM) survival curves were generated for visualization using GraphPad Prism software (version 8.4.2). Furthermore, a stratified analysis was performed, and inter-study heterogeneity was evaluated using Chi-square Q-tests to explore the collective effect of NUGs on GC survival and potential interactions between NUGs and covariates.

A linear regression model was utilized to conduct eQTL analysis. The Kruskal-Wallis test, Dunn’s test, and the Wilcoxon rank-sum test were used to explore the correlation between gene expression and TNM stage. The Wilcoxon rank-sum test was also employed to compare stromal scores, immune scores, ESTIMATE scores, and enrichment scores of DCs and NK cells between low- and high-expression groups of corresponding genes. Moreover, the Spearman correlation test was applied to explore the correlation between the expression of two genes in the TCGA dataset of 408 STAD tissues, as well as to examine the relationship between gene expression and the infiltration of immune cells. All statistical analyses were conducted using SAS software 9.4 (SAS Institute, Cary, NC, USA) and R software (version 3.6.1 and 4.4.1, R Foundation, Vienna, Austria). A two-tailed *P-*value < 0.05 was considered statistically significant.

## Results

### Associations between SNPs in NK cell-related genes and GC survival

**Figure 1:**
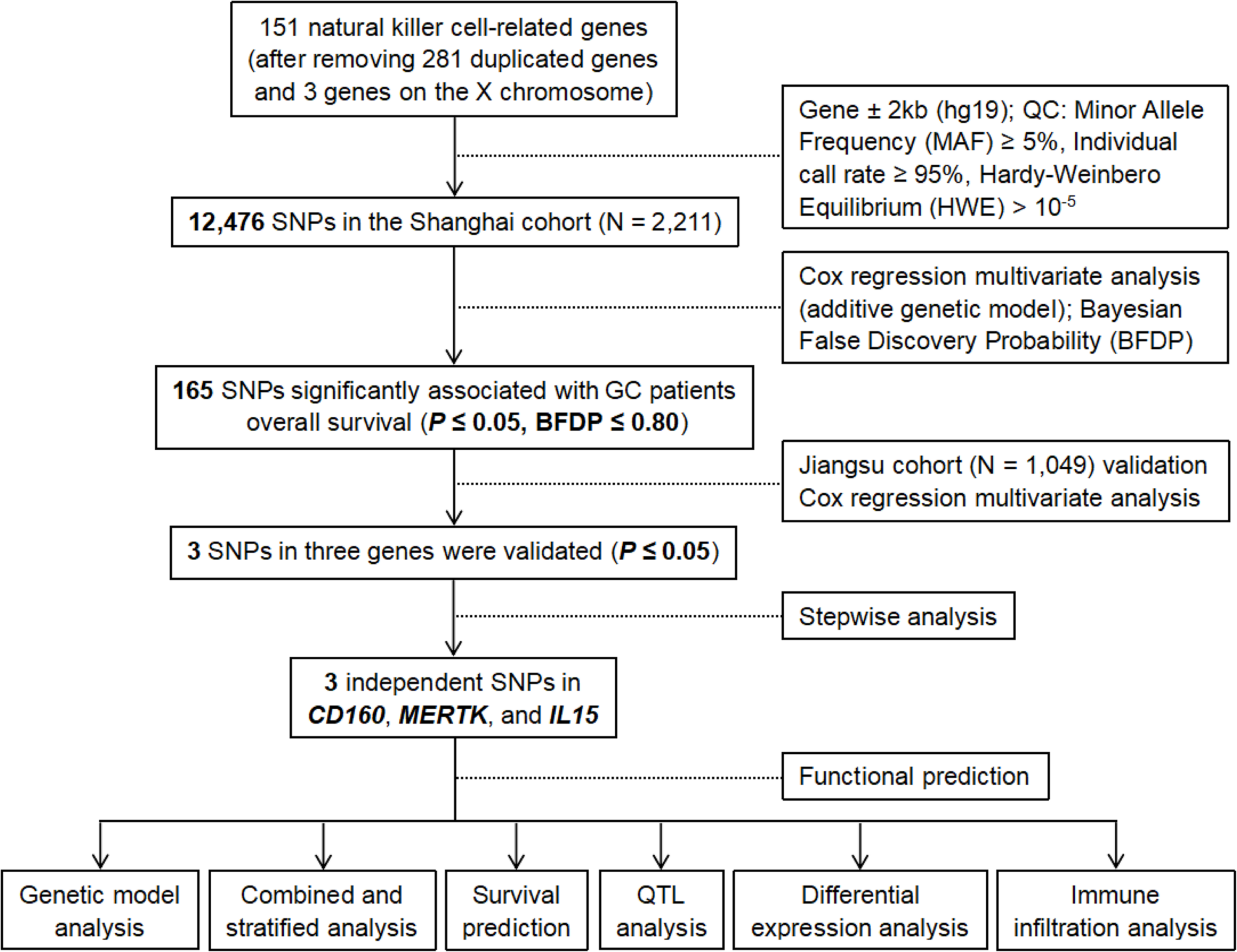
The flowchart of the present study. GC, gastric cancer; QC, quality control; QTL, quantitative trait loci; SNP, single nucleotide polymorphism.

The study flowchart is shown in **Figure 1**. Among the 12,476 SNPs in 151 NK cell-related genes, 165 SNPs were found to be significantly associated with the OS of GC patients in the single-locus analysis in the Shanghai cohort (*P*_adj_ ≤ 0.05 and BFDP ≤ 0.80). These significant SNPs were subsequently replicated in the Jiangsu cohort, with three SNPs in three genes remaining significant (i.e., *CD160* rs9728526 A>G, *MERTK* rs114788905 G>A, and *IL15* rs140007893 T>A) (*P*_adj_ ≤ 0.05).

### Associations between independent SNPs and GC survival in the Shanghai cohort

As shown in **Table 1**, the three SNPs mentioned above were independently associated with OS in GC following a stepwise analysis (*P* = 0.006, *P* = 0.033, and *P* = 0.028, respectively). Additionally, age at diagnosis, TNM stage, and chemotherapy were also found to be independently associated with OS (all *P* < 0.001). The consistent results of the meta-analysis for these three independent SNPs across the Shanghai and Jiangsu cohorts are presented in **Table 2**, with no heterogeneity observed. Additionally, Manhattan plots (**Supplementary Figure 1**) and regional association plots (**Supplementary Figure 2**) were generated to visualize the locations of these three identified SNPs.

**Table 1:** Three independent SNPs were identified in the multivariable Cox proportional hazards regression analysis in the Shanghai cohort (N = 2,211).

Variables	Category	Frequency (%)	Deaths (%)	HR (95% CI) ^a^	*P* ^a^
Age at diagnosis	< 60	1,028 (46.49)	244 (23.74)	1.00	
	≥ 60	1,183 (53.51)	500 (42.27)	1.78 (1.52-2.08)	<0.001
Sex	Female	646 (29.22)	212 (32.82)	1.00	
	Male	1,565 (70.78)	532 (33.99)	0.97 (0.81-1.16)	0.741
Smoking status	Never	1,424 (64.41)	468 (32.87)	1.00	
	Current	130 (5.88)	85 (65.38)	2.47 (1.93-3.17)	<0.001
	Former	657 (29.72)	191 (29.07)	0.85 (0.69-1.03)	0.100
Drinking status	No	1,636 (73.99)	556 (33.99)	1.00	
	Yes	575 (26.01)	188 (32.70)	0.96 (0.79-1.16)	0.665
TNM stage	I	646 (29.22)	41 (6.35)	1.00	
	II	564 (25.51)	120 (21.28)	4.98 (3.43-7.21)	<0.001
	III	1,001 (45.27)	583 (58.24)	18.71 (13.32-26.28)	<0.001
Chemotherapy	No	818 (37.00)	200 (24.45)	1.00	
	Yes	1,393 (63.00)	544 (39.05)	0.59 (0.49-0.70)	<0.001
Radiotherapy	No	2,109 (95.39)	693 (32.86)	1.00	
	Yes	102 (4.61)	51 (50.00)	1.17 (0.88-1.56)	0.277
***CD160* rs9728526 A>G**	**AA/AG/GG**	**1,066 (48.21)/946 (42.79)/199 (9.00)**	**353 (33.11)/306 (32.35)/85 (42.71)**	**1.16 (1.05-1.29)**	**0.006**
***MERTK* rs114788905 G>A**	**GG/GA/AA**	**566 (25.60)/1,120 (50.66)/525 (23.74)**	**206 (36.40)/383 (34.20)/155 (29.52)**	**0.89 (0.81-0.99)**	**0.033**
***IL15* rs140007893 T>A**	**TT/TA/AA**	**1,948 (88.10)/255 (11.53)/8 (0.36)**	**667 (34.24)/76 (29.80)/1 (12.50)**	**0.77 (0.62-0.97)**	**0.028**

CI, confidence interval; HR, hazards ratio; SNP, single nucleotide polymorphism; TNM, tumor-node-metastasis.

^a^ Stepwise analysis included age at diagnosis, sex, smoking status, drinking status, TNM stage, chemotherapy, radiotherapy, PC7, PC8, and SNPs. Footnote: The results for independent SNPs are shown in bold.

**Table 2:** Associations of three independent SNPs with overall survival in both discovery and validation datasets.

SNP	Allele ^a^	Gene	Shanghai cohort (N = 2,211)				Jiangsu cohort (N = 1,049)			Meta-analysis (N = 3,260)			
			BFDP	EAF	HR (95% CI) ^b^	*P* ^b^	EAF	HR (95% CI) ^c^	*P* ^c^	HR (95% CI) ^d^	*P* ^d^	*P*_het_ ^e^	I^2^
rs9728526	A>G	*CD160*	0.45	0.30	1.18 (1.06-1.31)	0.003	0.31	1.16 (1.01-1.33)	0.031	1.17 (1.08-1.28)	2.1×10^-4^	0.847	0
rs114788905	G>A	*MERTK*	0.79	0.49	0.88 (0.79-0.97)	0.014	0.47	0.83 (0.72-0.94)	0.005	0.86 (0.79-0.93)	3.0×10^-4^	0.494	0
rs140007893	T>A	*IL15*	0.77	0.06	0.76 (0.61-0.96)	0.019	0.06	0.73 (0.54-0.97)	0.033	0.75 (0.63-0.90)	0.002	0.831	0

BFDP, Bayesian False Discovery Probability; CI, confidence interval; EAF, effect allele frequency; HR, hazard ratio; SNP, single nucleotide polymorphism.

^a^ Reference allele > effect allele.

^b^ Adjusted for age at diagnosis, sex, smoking status, drinking status, TNM stage, chemotherapy, radiotherapy, PC7, and PC8.

^c^ Adjusted for age at diagnosis, sex, TNM stage, tumor location, chemotherapy or radiotherapy, and top ten PCs.

^d^ Meta-analysis in the fix-effects model.

^e^ *P*_het_: *P* value for heterogeneity by Cochrane’s Q test.

As shown in **Table 3**, the additive models indicated that the *CD160* rs9728526 G allele was associated with poor OS (*P*_trend_ = 0.003) and shorter five- and ten-year RMST, whereas both the *MERTK* rs114788905 A allele and the *IL15* rs140007893 A allele were associated with better OS (*P*_trend_ = 0.014 and 0.019, respectively) and longer five- and ten-year RMST. Similar results were observed in the dominant models for *CD160* rs9728526 and *IL15* rs140007893, as well as in the recessive model for *MERTK* rs114788905. The KM survival curves for these findings are shown in **Supplementary Figure 3**.

**Table 3:** Associations between three independent SNPs and survival of GC in the Shanghai cohort (N = 2,211).

Genotype	Frequency	OS ^a^			Five-year RMST (95% CI)		*P* ^a,b^	Ten-year RMST (95% CI)	*P* ^a,b^	Log-rank *P*
		Deaths (%)	HR (95% CI)	*P*						
*CD160* rs9728526 A>G										0.006
AA	1,066	353 (33.11)	1.00		50.2 (49.1-51.2)			91.3 (88.7-93.9)		
AG	946	306 (32.35)	1.16 (1.00-1.36)	0.054	50.4 (49.3-51.5)		0.069	92.0 (89.3-94.8)	0.003
GG	199	85 (42.71)	1.40 (1.10-1.77)	0.006	45.8 (43.0-48.6)		<0.001	81.2 (74.5-87.8)	<0.001
Trend test				0.003					
Dominant model										0.498
AA	1,066	353 (33.11)	1.00		50.2 (49.1-51.2)			91.3 (88.7-93.9)		
AG+GG	**1,145**	**391 (34.15)**	**1.21 (1.05-1.40)**	**0.011**	49.6 (48.5-50.6)		<0.001	90.2 (87.6-92.7)	<0.001
*MERTK* rs114788905 G>A										0.031
GG	566	206 (36.40)	1.00		49.2 (47.7-50.7)			88.1 (84.4-91.7)		
GA	1,120	383 (34.20)	0.95 (0.80-1.13)	0.574	49.3 (48.3-50.4)		0.145	89.9 (87.3-92.5)	<0.001
AA	525	155 (29.52)	0.77 (0.62-0.94)	0.012	51.8 (50.4-53.2)		<0.001	95.1 (91.6-98.7)	<0.001
Trend test				0.014					
Recessive model										0.014
GG+GA	1,686	589 (34.93)	1.00		49.3 (48.4-50.2)			89.3 (87.2-91.4)		
AA	525	155 (29.52)	0.79 (0.66-0.94)	0.010	51.8 (50.4-53.2)		<0.001	95.1 (91.6-98.7)	<0.001
Or reverse										
AA	525	155 (29.52)	1.00		51.8 (50.4-53.2)			95.1 (91.6-98.7)		
GG+GA	**1,686**	**589 (34.93)**	**1.27 (1.06-1.51)**	**0.010**	49.3 (48.4-50.2)		<0.001	89.3 (87.2-91.4)	<0.001
*IL15* rs140007893 T>A										0.174
TT	1,948	667 (34.24)	1.00		49.7 (48.9-50.5)			90.2 (88.3-92.2)		
TA	255	76 (29.80)	0.80 (0.63-1.02)	0.067	51.2 (49.2-53.2)		<0.001	93.9 (88.8-99.0)	<0.001
AA	8	1 (12.50)	0.22 (0.03-1.56)	0.129	55.5 (47.3-63.7)		<0.001	108.0 (86.0-130.0)	<0.001
Trend test				0.019					
Dominant model										0.102
TT	1,948	667 (34.24)	1.00		49.7 (48.9-50.5)			90.2 (88.3-92.2)		
TA+AA	263	77 (29.28)	0.77 (0.61-0.98)	0.033	51.3 (49.4-53.3)		<0.001	94.3 (89.3-99.4)	<0.001
Or reverse										
TA+AA	263	77 (29.28)	1.00		51.3 (49.4-53.3)			94.3 (89.3-99.4)		
TT	**1,948**	**667 (34.24)**	**1.30 (1.02-1.64)**	**0.033**	49.7 (48.9-50.5)		<0.001	90.2 (88.3-92.2)	<0.001
NUG ^c^										0.036
0	28	7 (25.00)	1.00		53.2 (48.3-58.0)			97.7 (83.3-112.1)		
1	354	99 (27.97)	1.53 (0.71-3.30)	0.283	52.3 (50.6-53.9)		<0.001	96.6 (92.4-100.8)	0.001
2	1,062	366 (34.46)	2.28 (1.08-4.85)	0.031	49.6 (48.6-50.7)		<0.001	89.9 (87.3-92.6)	<0.001
3	767	272 (35.46)	2.42 (1.14-5.14)	0.022	49.0 (47.7-50.3)		<0.001	88.8 (85.6-92.0)	<0.001
Trend test				<0.001					
Dichotomized NUG										0.004
0-1	382	106 (27.75)	1.00		52.3 (50.8-53.9)			96.7 (92.6-100.7)		
2-3	1,829	638 (34.88)	1.59 (1.29-1.95)	<0.001	49.4 (48.5-50.2)		<0.001	89.5 (87.4-91.5)	<0.001

CI, confidence interval; GC, gastric cancer; HR, hazard ratio; NUG, number of unfavorable genotype; OS, overall survival; RMST, restricted mean survival time; SNP, single nucleotide polymorphism.

^a^ Adjust age at diagnosis, sex, smoking status, drinking status, TNM stage, chemotherapy, radiotherapy, PC7, and PC8.

^b^ The *P*-value for the difference in the RMST subtraction between two groups.

^c^ Unfavorable genotypes were *CD160* rs9728526 AG+GG, *MERTK* rs114788905 GG+GA, and *IL15* rs140007893 TT, and their results are in bold.

### Combined analysis of three independent SNPs on GC survival in the Shanghai dataset

**Figure 2:**
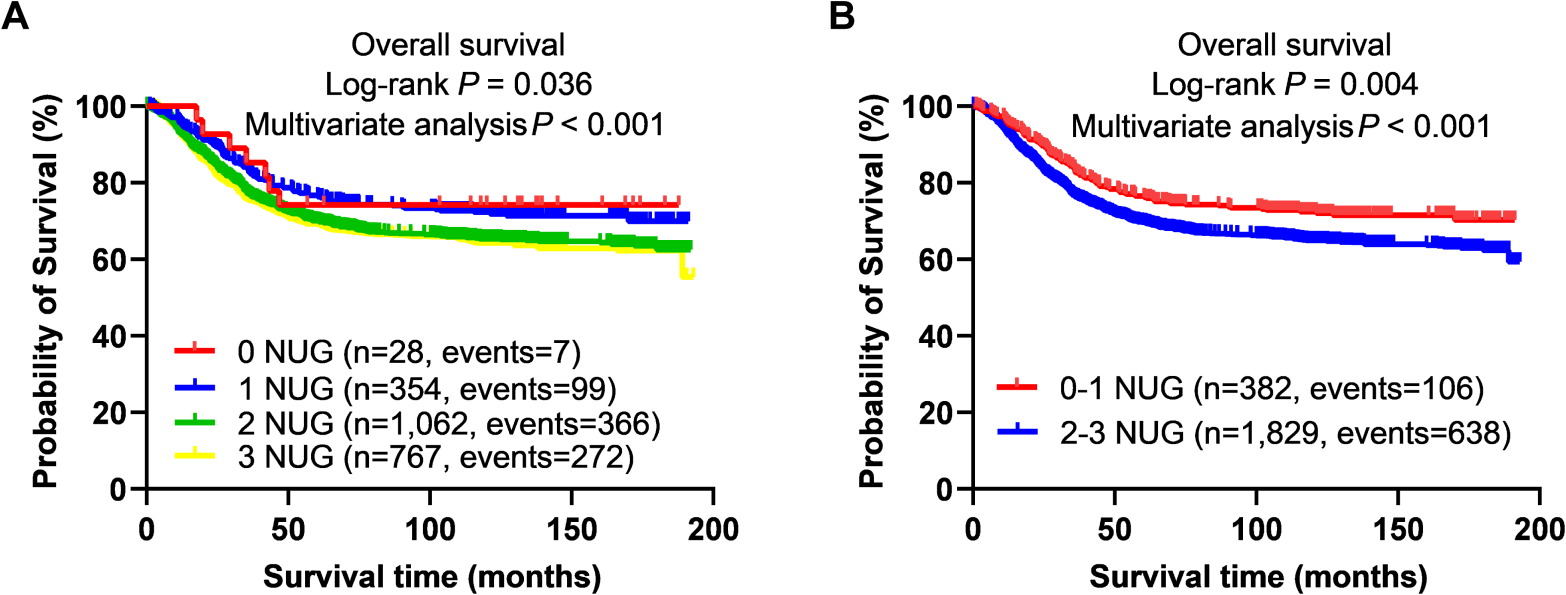
Prediction of GC survival with combined unfavorable genotypes. KM survival curves in the Shanghai GWAS dataset for **(A)** OS with the combined unfavorable genotypes; **(B)** OS with the dichotomized groups of the NUGs. #Unfavorable genotypes were CD160 rs9728526 AG+GG, MERTK rs114788905 GG+GA, and IL15 rs140007893 TT. GC, gastric cancer; GWAS, genome-wide association study; KM, Kaplan–Meier; NUGs, number of unfavorable genotypes; OS, overall survival.

The unfavorable genotypes (i.e., rs9728526 AG+GG, rs114788905 GG+GA, and rs140007893 TT) were combined into a genetic score, which was used to categorize all patients into four groups (i.e., 0, 1, 2, and 3). As shown in **Table 3**, a higher NUG score was associated with worse OS (*P*_trend_ < 0.001) and shorter five- and ten-year RMST (all *P* < 0.05). Furthermore, compared with the 0–1 NUG group, the 2–3 NUG group exhibited significantly poorer OS and shorter RMST (all *P* < 0.05). The KM survival curves for these findings are shown in **Figure 2**.

### Stratified analysis for the effect of NUG on GC survival

As shown in **Supplementary Table 4**, stratified analysis demonstrated that, compared to the 0–1 NUG group, the 2–3 NUG group had significantly worse OS across various subgroups, including patients aged ≥ 60 years at diagnosis, females, males, never smokers, current smokers, non-drinkers, TNM stage III, with chemotherapy, without chemotherapy, and no radiotherapy (all *P* < 0.05). Additionally, the interaction between NUG and TNM stage was statistically significant (*P*_inter_ = 0.047).

### Time-dependent AUC and ROC curves to evaluate the prognostic value of SNPs

As shown in **Supplementary Figure 4**, the 5-year AUC for the three independent SNPs was 0.541, which is lower than the AUCs of 0.577 for chemotherapy, 0.586 for age at diagnosis, and 0.780 for TNM stage. Similarly, the 10-year AUC for the three independent SNPs was 0.542, also lower than the AUCs of 0.556 for chemotherapy, 0.633 for age at diagnosis, and 0.756 for TNM stage. Additionally, after incorporating the three independent SNPs into the prognostic model, OS prediction was significantly enhanced, with the C-index increasing from 0.779 to 0.782 (*P* = 0.025). Moreover, the NRI for 5-year and 10-year survival increased by 1.4% (95% CI: -0.8%-3.7%) and 3.3% (95% CI: 1.0%-5.3%), respectively. The IDI for 5-year and 10-year survival significantly improved by 0.6% (95% CI: 0.1%-1.4%, *P* = 0.010) and 0.7% (95% CI: 0.1%-1.5%, *P* = 0.016), respectively (**Supplementary Table 5**).

We further performed time-dependent AUC and ROC curve analyses for OS at the 12th, 36th, 60th, 96th, and 120th months in the Shanghai cohort to assess the prognostic value of these three SNPs. The time-dependent AUC for OS prediction is shown in **Supplementary Figure 5A**. The AUC showed no significant increase at the 12th, 36th, and 60th months, but was significantly improved at the 96th month (*P* = 0.034) and the 120th month (*P* = 0.035) (**Supplementary Figure 5**). These findings suggest that these SNPs possess certain prognostic value. However, given the relatively low values of AUC, NRI, and IDI, integrating more possible SNPs with subtle effects into a polygenic risk score will be necessary to enhance predictive performance in future studies.

### Functional annotation of independent SNPs

The functional prediction results for the three independent SNPs across five bioinformatics tools are provided in **Supplementary Table 6**. rs9728526 is an upstream variant of *CD160* that affects transcription factor binding sites (TFBSs) and is located within DNase I hypersensitivity sites, which are considered potential regulatory regions. ChIP-sequencing data from the HaploReg v4.2 database suggest that rs9728526 is involved in promoter function in stomach tissues (**Supplementary Figures 6A, B**). rs114788905 is located in the tenth intron of *MERTK*, overlaps potential enhancer and promoter regions marked by H3K4me1, H3K4me3, and H3K27ac, as well as in DNase I hypersensitivity sites. ChIP-sequencing data from the HaploReg v4.2 database further imply that rs114788905 is involved in promoter function in stomach tissues (**Supplementary Figure 6C, D**). rs140007893 is an intronic variant located in the second intron of *IL15* (**Supplementary Figure 6E**).

Given that all three SNPs were associated with alterations in TFBS motifs, allele-specific transcription factor binding was explored using the JASPAR database. The *CD160* rs9728526 G allele may influence the binding motifs of FEZF2, KLF2, TCF3, and TCF12; the *MERTK* rs114788905 A allele may affect ZXDB binding; and the *IL15* rs140007893 A allele may impact HMBOX1 binding (all relative scores ≥ 90%) (**Supplementary Figures 7A–F**). Correlation analyses in 408 STAD tissues revealed significant correlations between the expression of *CD160* and that of *FEZF2* and *TCF12*, *MERTK* and *ZXDB*, as well as *IL15* and *HMBOX1* (**Supplementary Figures 7G–I**). No significant correlations were observed between *CD160* expression and the expression of *KLF2* and *TCF3* (**Supplementary Figure 7G**). These results suggest that the SNPs may regulate gene expression through allele-specific TF binding.

### The results of QTL analyses

**Figure 3:**
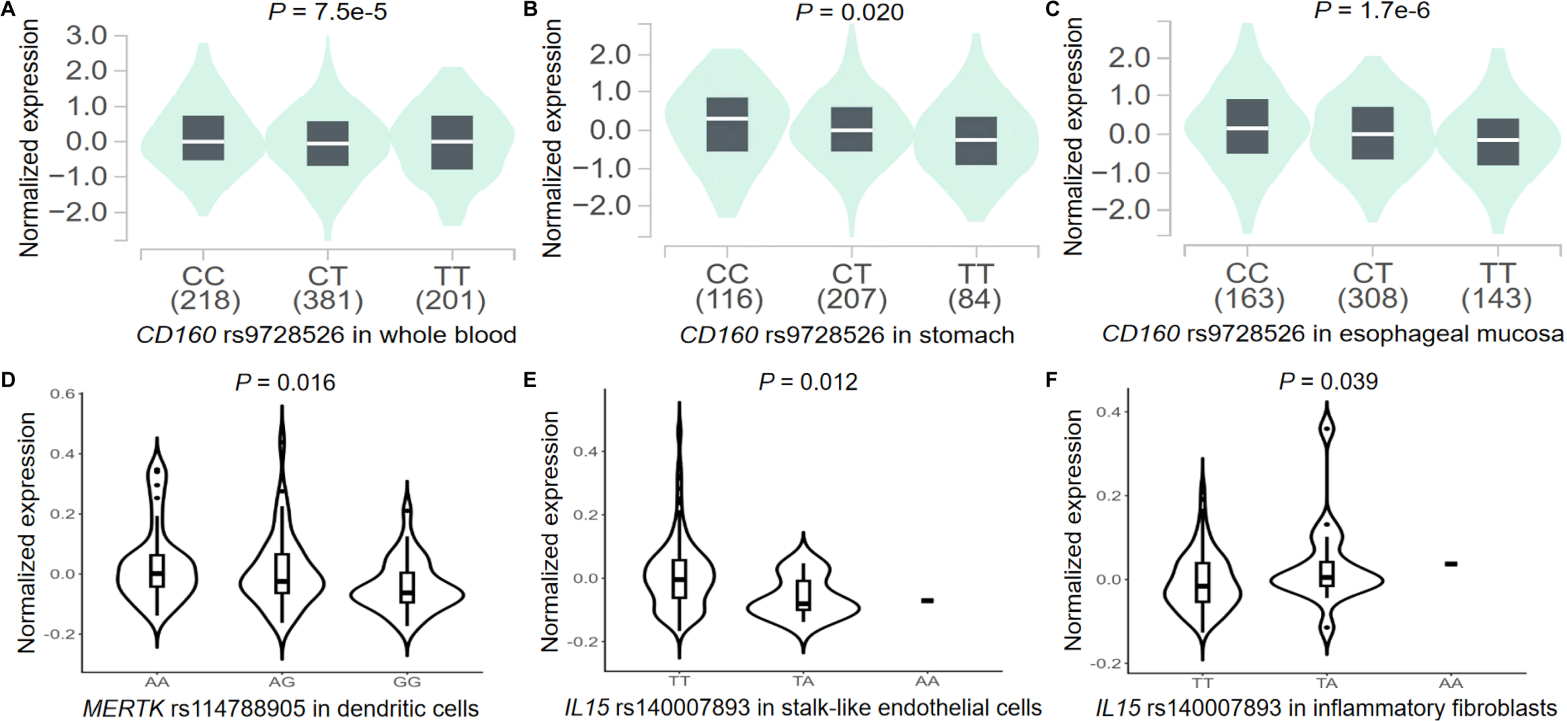
The results of the eQTL analyses for three independent SNPs. The CD160 rs9728526 G allele was significantly associated with higher mRNA expression levels of CD160 in **(A)** whole blood samples, **(B)** normal stomach tissues, and **(C)** normal esophageal mucosa tissues from the GTEx project; **(D)** single-cell eQTL analysis indicated that the MERTK rs114788905 A allele was significantly associated with higher mRNA expression levels of MERTK in gastric dendritic cells from the scGaTE website (N=203); single-cell eQTL analysis showed that the IL15 rs140007893 A allele was significantly associated with lower IL15 mRNA expression in **(E)** gastric stalk-like endothelial cells and higher expression in **(F)** gastric inflammatory fibroblasts from the scGaTE website (N=203). eQTL, expression quantitative trait loci; GTEx, Genotype-Tissue Expression.

We first performed eQTL analyses to explore the correlations between genotypes and gene expression. Data from the GTEx project indicated that the *CD160* rs9728526 G allele was significantly associated with higher mRNA expression levels of *CD160* in whole blood (*P* = 7.5e-5, **Figure 3A**), normal stomach tissues (*P* = 0.020, **Figure 3B**), and normal esophageal mucosa tissues (*P* = 1.7e-6, **Figure 3C**), whereas the *MERTK* rs114788905 A allele showed no significant correlation (**Supplementary Figures 8A–C**). Meta-analysis data from the eQTLGen database revealed that the *CD160* rs9728526 G allele and *MERTK* rs114788905 A allele were associated with higher mRNA expression levels of *CD160* and *MERTK* in whole blood, respectively (**Supplementary Figure 8D**). Existing data from 1000G indicated no significant association between the genotypes of the three independent SNPs and mRNA expression levels of the corresponding genes in European and East Asian individuals across additive, dominant, and recessive models (**Supplementary Figure 9**).

Moreover, available and significant sc-eQTL data from the scGaTE platform showed that the *MERTK* rs114788905 A allele was associated with higher mRNA expression levels of *MERTK* in gastric DCs (*P* = 0.016, **Figure 3D**). The *IL15* rs140007893 A allele was associated with lower mRNA expression levels of *IL15* in gastric stalk-like endothelial cells (*P* = 0.012, **Figure 3E**), while it was also associated with higher mRNA expression levels of *IL15* in gastric inflammatory fibroblasts (*P* = 0.039, **Figure 3F**). Non-significant gastric sc-eQTL results for *MERTK* rs114788905 and *IL15* rs140007893 are presented in **Supplementary Table 7**. Furthermore, as shown in **Supplementary Table 8**, additional sc-eQTL analyses across diverse immune cell types indicated that the *CD160* rs9728526 G allele was significantly associated with higher *CD160* expression in CD8^+^ T cells, memory CD4^+^ T cells, and memory T cells (all *P* < 0.05). The *MERTK* rs114788905 A allele was associated with higher mRNA expression levels of *MERTK* in naïve monocytes, while it was associated with lower *MERTK* expression in memory Th1 cells, memory Th2 cells, naïve CD4^+^ T cells, and naïve regulatory T cells (Treg cells) (all *P* < 0.05). The *IL15* rs140007893 A allele was associated with lower *IL15* expression in memory regulatory T cells (Treg cells) and several types of monocytes (all *P* < 0.05).

Subsequently, sQTL analyses from the FIVEx platform demonstrated that all three SNPs affected alternative splicing of their respective genes across blood, stomach, and immune cell types (**Supplementary Table 9**). Lastly, we performed mQTL and bQTL analyses on *CD160* rs9728526, and mQTL and hQTL analyses on *MERTK* rs114788905 using the QTLbase database. As shown in **Supplementary Figure 10A**, rs9728526 was significantly associated with methylation of various CpG sites across a wide range of specific cell types and tissues (all *P* < 0.05), as well as with the binding status of the transcription factor NF-kB in lymphocytes (*P* = 0.004, **Supplementary Figure 10B**). Similarly, rs114788905 was significantly associated with methylation of cg08279316 in CD14^+^ monocytes (*P* = 0.003, **Supplementary Figure 10C**). The hQTL analyses showed that rs114788905 was significantly associated with histone modifications of H3K4me1 in naïve CD4^+^ T cells and H3K27ac in CD14^+^ monocytes (all *P* < 0.05, **Supplementary Figure 10D**). The bQTL, hQTL, and mQTL data for *IL15* rs140007893 were not available in the QTLbase database.

### The analysis of differential expression and survival in GC

**Figure 4:**
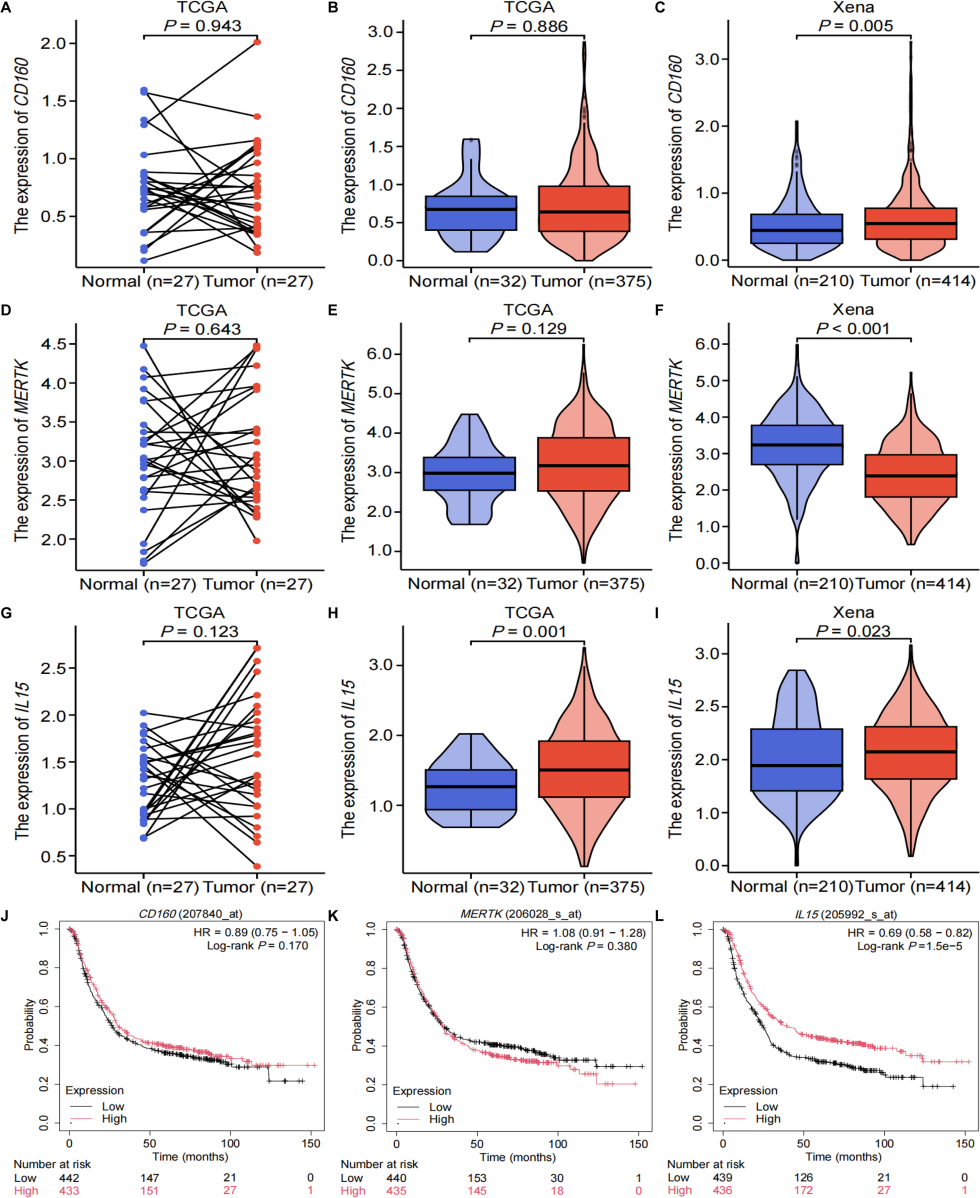
The results of differential mRNA expression and survival analyses for SNP-associated genes in GC. The paired **(A)** and unpaired tests **(B)** indicated no significant difference in CD160 mRNA expression levels between GC and adjacent normal tissues based on TCGA omics data; **(C)** CD160 mRNA expression levels were upregulated in GC in the Xena database; the paired **(D)** and unpaired tests **(E)** showed no significant difference in MERTK mRNA expression levels between GC and adjacent normal tissues based on TCGA omics data; **(F)** data from the Xena database indicated that MERTK mRNA expression levels were downregulated in GC; **(G)** the paired test indicated no significant difference in IL15 mRNA expression levels between GC and adjacent normal tissues, **(H)** while unpaired tests showed elevated IL15 expression in GC based on TCGA omics data; **(I)** data from the Xena database demonstrated that IL15 mRNA expression levels were upregulated in GC; the expression levels of **(J)** CD160 and **(K)** MERTK were not associated with overall survival in GC; **(L)** high mRNA expression levels of IL15 were associated with better overall survival in GC. GC, gastric cancer; HR, hazard ratio.

Omics data from the TCGA and the UCSC Xena databases were used to explore the expression of SNP-associated genes in GC. TCGA data showed no significant difference in the mRNA expression levels of *CD160* and *MERTK* between normal gastric tissues and GC tumor tissues (**Figures 4A–D**). However, unpaired tests with a larger sample size showed that *CD160* mRNA expression was upregulated, while *MERTK* mRNA expression was downregulated in GC using Xena data (**Figures 4C and 4F**). Additionally, the paired test indicated no significant difference in *IL15* mRNA expression levels between GC and adjacent normal tissues, while unpaired tests demonstrated elevated *IL15* expression in GC based on TCGA and Xena data (**Figures 4G–I**). Moreover, KM Plotter analysis showed that the mRNA expression levels of *CD160* and *MERTK* was not associated with OS in GC, while high mRNA expression levels of *IL15* were associated with favorable OS in GC (**Figures 4J–L**).

Furthermore, we explored the relationship between gene expression and TNM stage in GC. The results showed that the expression of *CD160* and *MERTK* was not associated with TNM stage (**Supplementary Figures 11A–C**). Compared to the TNM stage I group, *IL15* expression was significantly higher in the TNM stage III group (*P* = 0.007**, Supplementary Figure 11A**). Similarly, *IL15* expression was significantly elevated in the TNM stage III-IV group compared to the TNM stage I-II group (*P* = 0.021, **Supplementary Figure 11B**). However, no significant difference in *IL15* expression levels was observed between the TNM stage I-III and IV groups (**Supplementary Figure 11C**).

### Enrichment analysis

To further investigate the influence of *CD160*, *MERTK*, and *IL15* expression on specific biological processes and functions, we performed GO enrichment analyses and GSEA using the CAMOIP database in STAD patients. We found that *CD160* was involved in various biological processes related to gene expression, with elevated *CD160* expression negatively correlated with rRNA processing (**Supplementary Figures 12A, B**). Moreover, both *MERTK* and *IL15* were involved in various immune-related biological pathways (**Supplementary Figures 12C, E**). The results of GSEA showed that elevated expression of *MERTK* was positively correlated with the adaptive immune response (**Supplementary Figure 12D**), while high *IL15* expression was positively correlated with lymphocyte-mediated immunity (**Supplementary Figure 12F**) (all accessed on September 12, 2025).

### Immune infiltration analyses

**Figure 5:**
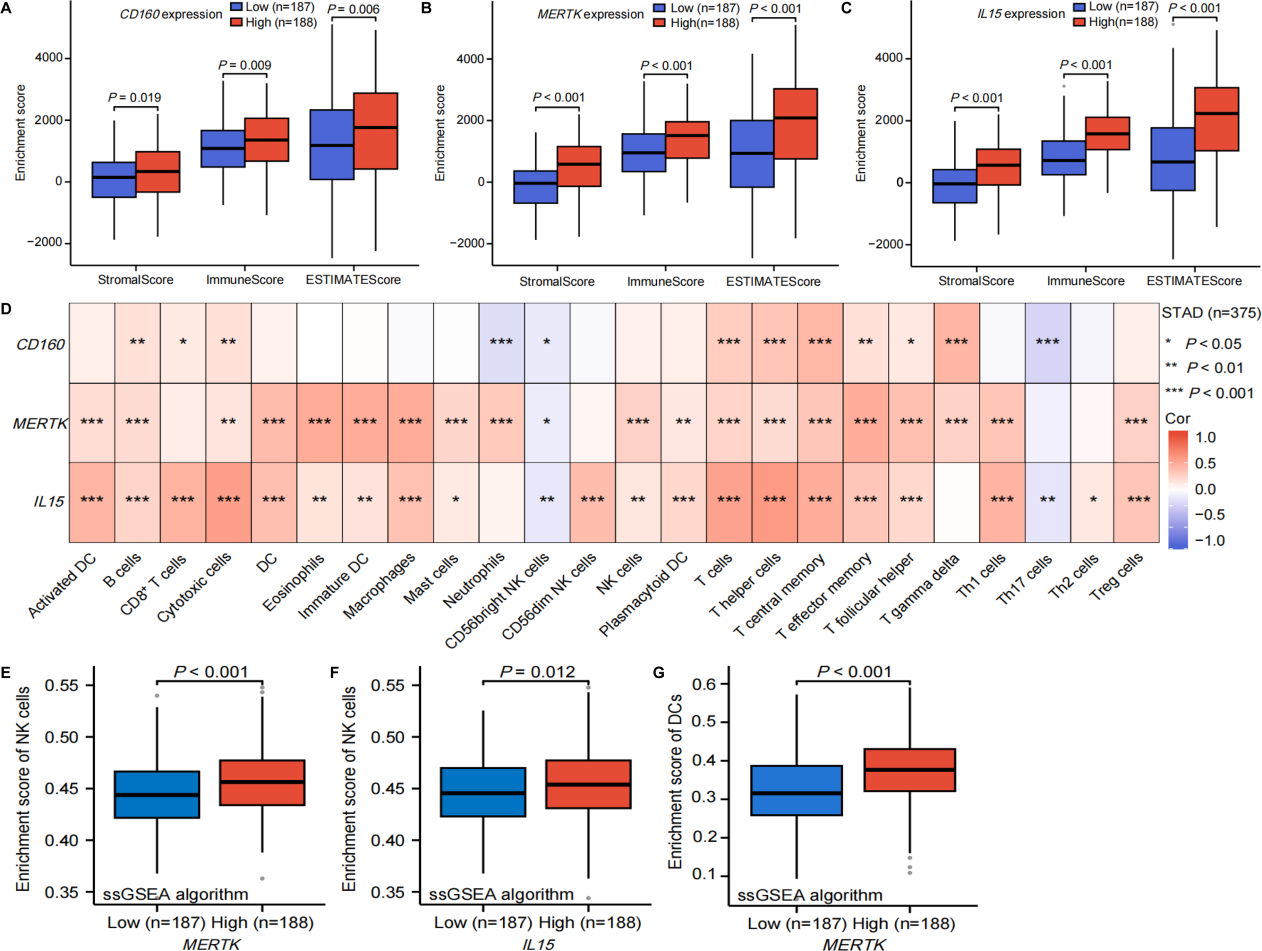
The results of immune infiltration analyses in GC. The box plots showed the significant differences in stromal score, immune score, and ESTIMATE score between the low- and high-expression groups of **(A)** CD160, **(B)** MERTK, and **(C)** IL15 using the ESTIMATE algorithm; **(D)** the heatmap presented the correlation between CD160, MERTK, and IL15 expression and the infiltration levels of 24 immune cell types using data from the TCGA database, with the ssGSEA algorithm and Spearman correlation test; the box plots showed significant differences in NK cells enrichment score between the low- and high-expression groups of **(E)** MERTK and **(F)** IL15 using the ssGSEA algorithm; the violin plot demonstrated significant differences in enrichment score of DCs between the low- and high-expression groups of **(G)** MERTK using the ssGSEA algorithm. DC, dendritic cell; IPS, immunophenotypic score; NK cell, natural killer cell; ssGSEA, single-sample gene set enrichment analysis; STAD, stomach adenocarcinoma; TCGA, the Cancer Genome Atlas; Treg cells, regulatory T cells.

Immune infiltration analyses were conducted to explore the potential impact of SNP-associated gene expression on the STAD TME. We found that the stromal, immune, and ESTIMATE scores were significantly higher in the *CD160* high-expression group compared to the low-expression group (**Figure 5A**). Similar trends were observed for *MERTK* (**Figure 5B**) and *IL15* (**Figure 5C**). Meanwhile, elevated *CD160* expression was positively correlated with increased abundance of both stromal and immune cells (**Supplementary Figure 13A**). Similar trends were observed for *MERTK* (**Supplementary Figure 13B**) and *IL15* (**Supplementary Figure 13C**). Further analyses based on IPS revealed that the *CD160* high-expression group was associated with higher effector cell scores and lower immune checkpoint molecules scores (**Supplementary Figure 13D**), the *MERTK* high-expression group was associated with higher effector cells scores and lower scores of suppressor cells and immune checkpoint molecules (**Supplementary Figure 13E**), and the *IL15* high-expression group was associated with higher scores of MHC molecules and effector cells and lower scores of suppressor cells and checkpoint molecules compared to the corresponding low-expression groups (**Supplementary Figure 13F**). Collectively, these findings imply that high expression of *CD160*, *MERTK*, and *IL15* is correlated with an active immune microenvironment in GC.

As detailed in **Figure 5D**, we further found that the expression of *CD160*, *MERTK*, and *IL15* was significantly correlated with the infiltration of numerous immune cell types. Specifically, *CD160* expression was not correlated with NK cell infiltration, consistent with the findings from the TISIDB database (**Supplementary Figure 13G**). Notably, *CD160* expression was significantly negatively correlated with CD56bright NK cell infiltration (**Figure 5D**). Both *MERTK* and *IL15* expression were positively correlated with NK cell infiltration, which was validated by data from the TISIDB database (**Supplementary Figures 13H and 13I**). Furthermore, no difference was observed in the enrichment score of NK cells between the low- and high-expression groups of *CD160* (**Supplementary Figure 13J**), while the enrichment score of NK cells was significantly elevated in both the *MERTK* and *IL15* high-expression groups compared to the corresponding low-expression groups (**Figures 5E and 5F**).

Guided by the discovery from gastric sc-eQTL analyses, we further investigated the relationships between *MERTK* expression and DC infiltration, as well as *IL15* expression and the absolute abundance of endothelial cells and fibroblasts in GC. We found that *MERTK* expression was positively correlated with the infiltration of activated and immature DCs (**Figure 5D**), and the enrichment score of DCs was significantly upregulated in the *MERTK* high-expression group compared to the low-expression group (**Figure 5G**). The MCPcounter algorithm indicated no significant difference in the abundance of endothelial cells between the low- and high-expression groups of *IL15*, while the abundance of fibroblasts was significantly higher in the *IL15* high-expression group compared to the low-expression group (**Supplementary Figure 13K**). Taken together, the above findings suggest that *CD160*, *MERTK*, and *IL15* may affect immune and stromal cell infiltration within the GC TME.

## Discussion

Immunotherapies that stimulate or enhance the host immune system to recognize and eliminate cancer cells have improved survival in multiple solid tumors. Compared with chimeric antigen receptor (CAR)-engineered T (CAR-T) cell therapy, CAR-NK cell therapy offers greater safety and efficacy, making it a promising immunotherapy for GC (46). To our knowledge, this is the first study to examine the associations between SNPs in NK cell-related genes and survival outcomes in GC. We identified three independent SNPs (*CD160* rs9728526 A>G, *MERTK* rs114788905 G>A, and *IL15* rs140007893 T>A) that independently predicted OS in patients with TNM stage I-III GC. Functional analyses revealed that these variants influenced gene expression and splicing patterns in specific tissues or immune cell types, together with alterations in histone modifications, methylation status, and transcription factor binding. Furthermore, expression of these genes was further associated with an active immune response and increased infiltration of multiple immune cell infiltrations within the TME, providing novel insight into their potential influence on GC survival.

*CD160*, the CD160 molecule, located on chromosome 1q21.1, encodes an immunoglobulin superfamily protein predominantly expressed on circulating NK cells and subsets of T cells (e.g., γδ T cell). It exerts a dual role in immunity by both promoting NK cell activation and functional enhancement, and being associated with T cell exhaustion (47, 48). *CD160* dysregulation has been reported across multiple solid tumors (49), but its prognostic significance varies by cancer type and immune context. For example, in esophageal squamous cell carcinoma, *CD160* is overexpressed as a mechanism of immune evasion (50). However, elevated *CD160* expression has been associated with better survival in breast cancer, as well as in patients with lung adenocarcinoma receiving first-line anti-PD-1 immunochemotherapy (51, 52). These discrepancies underscore complex roles of *CD160* in tumor immunity and cancer progression. Previous studies have also suggested that *CD160* may play a role in regulating immune responses in GC (53).

In our study, we observed a significant increase in *CD160* mRNA expression in GC tissues, and the *CD160* rs9728526 G allele was consistently associated with higher *CD160* expression in whole blood, stomach, esophageal mucosa, and various T cell subsets, as well as with poorer OS. This variant also altered transcription factor binding and methylation status, suggesting an epigenetic regulatory effect. Interestingly, high *CD160* expression was negatively correlated with CD56bright NK cell in the GC TME. CD56bright NK cells possess immunoregulatory functions, inhibit cancer cell proliferation, and promote apoptosis by secreting multiple cytokines, including IFN-γ, TNF-a, and GM-CSF (54). However, no significant association was observed between *CD160* expression and GC OS, suggesting that the relationship between *CD160* expression and survival may be influenced by the overall immune microenvironment rather than being a simple linear association. Moreover, the observed discrepancy may reflect linkage effects. As a variant located 2 kb upstream of *CD160*, rs9728526 could potentially regulate the expression of multiple neighboring genes, including *ANKRD35*, *NUDT17*, *PDZK1*, *PIAS3*, and *RNF115* (according to GTEx project data). Such multi-gene regulatory effect may contribute to variations in inconsistent prognostic outcomes. Given the limited evidence on *CD160* in GC, future studies should thoroughly investigate the mechanistic chain linking rs9728526 to *CD160* expression and ultimately to clinical prognosis, as well as the immunoregulatory role of *CD160* across disease stages.

*MERTK*, MER tyrosine kinase, a proto-oncogene located on chromosome 2q13, encodes a receptor tyrosine kinase of the TAM family, which is critical for NK cell activation, differentiation, and macrophage-mediated immune suppression (55, 56). Overexpression of *MERTK* has been linked to tumor progression, therapeutic resistance, and poor prognosis in various cancers (57). To date, very limited studies have investigated the role of *MERTK* in GC. Previous reports indicated that *MERTK* promotes GC cell proliferation *in vitro* and is associated with poor OS in GC patients without preoperative chemotherapy; however, it showed no association with OS in GC patients with preoperative chemotherapy (58).

In this study, the *MERTK* rs114788905 A allele was associated with higher *MERTK* expression in blood and exhibited immune cell–specific eQTL effects—upregulating *MERTK* in gastric dendritic cells and blood monocytes, while downregulating it in certain blood T-cell subsets, and predicted better survival. In contrast, *MERTK* mRNA expression was downregulated in GC tumor tissues, and expression levels of *MERTK* were not associated with OS, likely reflecting heterogeneity due to differences in sample size or therapeutic background (58). This pattern may also reflect tissue- and cell-type specificity, as blood-based eQTL effects may not directly translate to heterogeneous GC tumor tissue, which may further contribute to the inconsistent findings. Immune infiltration analysis further indicated that higher *MERTK* expression correlated with increased DC and NK cell infiltration, suggesting that the functional impact of *MERTK* in GC may be largely dependent on the TME and specific immune cell populations. Collectively, these observations suggest that the association between rs114788905 and favorable survival may be context-dependent, warranting further validation.

*IL15*, interleukin 15, located on chromosome 4q31.21, encodes a cytokine that regulates NK- and T-cell activation, cytotoxicity, proliferation, and survival. Previous studies have indicated that *IL15* expression is upregulated in GC and may exert a tumor-suppressive role by enhancing the recruitment and activation of T and NK cells within the TME (59–61). However, other studies have revealed that *IL15* contribute to GC cell epithelial-mesenchymal transition and metastasis by increasing the Treg cell ratio and upregulating PD-1 expression (60). Collectively, these findings suggest that the role of *IL15* in GC is likely immune context-dependent.

In our study, *IL15* mRNA levels were markedly upregulated in GC tissues, which is consistent with previous studies (59), and were associated with higher TNM stage and better OS, indicating its possible immunostimulatory role. The *IL15* rs140007893 A allele was linked to improved survival and correlated with differential *IL15* expression across fibroblasts, endothelial cells, monocytes, and Treg cells, highlighting cell type-specific regulation. Furthermore, immune infiltration analysis suggested that *IL15* expression was associated with the abundance of NK cells and fibroblasts. Fibroblasts were found to secrete IL-8, which stimulates NK cell activity and facilitates NK cell-mediated lysis of cancer cells (62). This pattern, coupled with elevated *IL15* expression in fIbroblasts, implies that *IL15* variants may promote an anti-tumor immune milieu by modulating NK cell infiltration and fibroblast recruitment. Additionally, reduced *IL15* expression in endothelial and Treg cells may further suppress angiogenesis and immunosuppression (63–65), contributing to a favorable outcome.

Collectively, these results demonstrate that genetic variation in NK cell-related genes can shape the immune landscape of GC in a cell type-specific manner, influencing gene expression, immune infiltration, and patient survival. The three identified SNPs provide potential biomarkers for risk stratification, and integration of such SNP-based markers into prognostic models may enhance survival prediction.

This study also advances understanding of the immunogenetic architecture of GC prognosis. Future functional studies, such as CRISPR-based gene editing, single-cell transcriptomics, and multi-omics integration, are warranted to validate these associations and delineate their biological mechanisms. Nevertheless, several limitations should be acknowledged. First, clinical covariates such as GC histopathological type (intestinal vs. diffuse), differentiation grade, chemotherapy regimens, and radiotherapy details were unavailable in both GWAS datasets. Second, as this study included only Chinese Han patients, generalizability to other populations remains to be established. Finally, the detailed molecular mechanisms underlying the observed associations were not revealed in our study, and additional functional experiments are required to confirm the causal mechanisms.

In conclusion, this two-stage GWAS analysis of NK cell-related genes identified *CD160* rs9728526, *MERTK* rs114788905, and *IL15* rs140007893 as independent prognostic SNPs in GC. These variants could modulate gene expression and influence the immune microenvironmental, offering novel avenues for biomarker development in GC.

## Data Availability

The original contributions presented in the study are included in the article/**Supplementary Material**. Further inquiries can be directed to the corresponding authors.

## Glossary

AUC: area under the curve
bQTL: transcription factor binding quantitative trait loci
BFDP: Bayesian false discovery probability
CAR: chimeric antigen receptor
CD160: CD160 molecule
CI: confidence interval
DC: dendritic cell
eQTL: expression quantitative trait loci
GC: gastric cancer
FUSCC: Fudan University Shanghai Cancer Center
GEO: Gene Expression Omnibus
GO: Gene Ontology
GSEA: gene set enrichment analysis
GTEx: Genotype-Tissue Expression
GWAS: genome-wide association study
hQTL: histone modification quantitative trait loci
HWE: Hardy-Weinberg equilibrium
HR: hazard ratio
IDI: integrated discrimination improvement
IL15: interleukin 15
IPS: immunophenotypic score
KM: Kaplan-Meier
LD: linkage disequilibrium
MAF: minor allelic frequency
MERTK: MER proto-oncogene tyrosine kinase
MHC: major histocompatibility complex
mQTL: methylation quantitative trait loci
NK cell: natural killer cell
NRI: net reclassification improvement
NUG: number of unfavorable genotype
OS: overall survival
PC: Principal Component
QTL: quantitative trait loci
RMST: restricted mean survival time
RNA-seq: RNA-sequencing
ROC: receiver operating characteristic
sc-eQTL: single-cell eQTL
SNPs: single nucleotide polymorphisms
sQTL: splicing quantitative trait loci
ssGSEA: single-sample gene set enrichment analysis
STAD: stomach adenocarcinoma
TCGA: the Cancer Genome Atlas
TFBS: transcription factor binding site
TME: tumor microenvironment
TNM: tumor-node-metastasis
Treg cell: regulatory T cell.
