# Supplementary material for: Genetic variants of CD160, MERTK, and IL15 in natural killer cell-related pathway predict gastric cancer survival
Source: Front Immunol. 2026 Apr 15;17:1765825. doi: 10.3389/fimmu.2026.1765825 (PMC13125121; doi:10.3389/fimmu.2026.1765825)
Supplement: Supplementary file 1 [file DataSheet1.docx]

| **Supplementary Table 1.** Comparison of characteristics between the Shanghai cohort and the Jiangsu cohort. | | | | | | | | | | |
| --- | --- | --- | --- | --- | --- | --- | --- | --- | --- | --- |
| **Variables** | **Shanghai cohort** | | | |  | **Jiangsu cohort** | | | | ***P*** ^b^ |
|  | **Frequency (%)** | **Deaths (%)** | ***P*** ^a^ | **Log-rank *P*** |  | **Frequency (%)** | **Deaths (%)** | ***P*** ^a^ | **Log-rank *P*** |  |
| **Total** | **2,211** | **744 (33.65)** |  |  |  | **1,049** | **469 (44.71)** |  |  | <0.001 |
| Median follow-up time (month) | 108.4 |  |  |  |  | 98.3 |  |  |  |  |
| Age at diagnosis |  |  | <0.001 | <0.001 |  |  |  | 0.007 | <0.001 | <0.001 |
| < 60 | 1,028 (46.49) | 244 (23.74) |  |  |  | 373 (35.56) | 146 (39.14) |  |  |  |
| ≥ 60 | 1,183 (53.51) | 500 (42.27) |  |  |  | 676 (64.44) | 323 (47.78) |  |  |  |
| Sex |  |  | 0.595 | 0.648 |  |  |  | 0.086 | 0.096 | 0.001 |
| Female | 646 (29.22) | 212 (32.82) |  |  |  | 250 (23.83) | 100 (40.00) |  |  |  |
| Male | 1,565 (70.78) | 532 (33.99) |  |  |  | 799 (76.17) | 369 (46.18) |  |  |  |
| Smoking status |  |  | <0.001 | <0.001 |  | NA |  |  |  |  |
| Never | 1,424 (64.41) | 468 (32.87) |  |  |  |  |  |  |  |  |
| Current | 130 (5.88) | 85 (65.38) |  |  |  |  |  |  |  |  |
| Former | 657 (29.72) | 191 (29.07) |  |  |  |  |  |  |  |  |
| Drinking status |  |  | 0.573 | 0.545 |  | NA |  |  |  |  |
| No | 1,636 (73.99) | 556 (33.99) |  |  |  |  |  |  |  |  |
| Yes | 575 (26.01) | 188 (32.70) |  |  |  |  |  |  |  |  |
| Depth of invasion |  |  | <0.001 | <0.001 |  |  |  | <0.001 | <0.001 | <0.001 |
| T1 | 538 (24.33) | 41 (7.62) |  |  |  | 160 (15.25) | 15 (9.38) |  |  |  |
| T2 | 314 (14.20) | 47 (14.97) |  |  |  | 148 (14.11) | 41 (27.70) |  |  |  |
| T3 | 415 (18.77) | 140 (33.73) |  |  |  | 20 (1.91) | 8 (40.00) |  |  |  |
| T4a/4b | 944 (42.70) | 516 (54.66) |  |  |  | 721 (68.73) | 405 (56.17) |  |  |  |
| Lymph node metastasis |  |  | <0.001 | <0.001 |  |  |  | <0.001 | <0.001 | 0.020 |
| N0 | 897 (40.57) | 105 (11.71) |  |  |  | 374 (35.65) | 77 (20.59) |  |  |  |
| N1 | 397 (17.96) | 97 (24.43) |  |  |  | 210 (20.02) | 98 (46.67) |  |  |  |
| N2 | 404 (18.27) | 176 (43.56) |  |  |  | 225 (21.45) | 128 (56.89) |  |  |  |
| N3a/3b | 513 (23.20) | 366 (71.35) |  |  |  | 240 (22.88) | 166 (69.17) |  |  |  |
| TNM stage (AJCC 8th) |  |  | <0.001 | <0.001 |  |  |  | <0.001 | <0.001 | <0.001 |
| I | 646 (29.22) | 41 (6.35) |  |  |  | 232 (22.12) | 31 (13.36) |  |  |  |
| II | 564 (25.51) | 120 (21.28) |  |  |  | 182 (17.35) | 60 (32.97) |  |  |  |
| III | 1,001 (45.27) | 583 (58.24) |  |  |  | 635 (60.53) | 378 (59.53) |  |  |  |
| Chemotherapy |  |  | <0.001 | <0.001 |  | NA |  |  |  |  |
| No | 818 (37.00) | 200 (24.45) |  |  |  |  |  |  |  |  |
| Yes | 1,393 (63.00) | 544 (39.05) |  |  |  |  |  |  |  |  |
| Radiotherapy |  |  | <0.001 | <0.001 |  | NA |  |  |  |  |
| No | 2,109 (95.39) | 693 (32.86) |  |  |  |  |  |  |  |  |
| Yes | 102 (4.61) | 51 (50.00) |  |  |  |  |  |  |  |  |
| Chemotherapy/radiotherapy |  |  | <0.001 | <0.001 |  |  |  | <0.001 | 0.004 | 0.559 |
| No | 801 (36.23) | 190 (23.72) |  |  |  | 369 (35.18) | 137 (37.13) |  |  |  |
| Yes | 1,410 (63.77) | 554 (39.29) |  |  |  | 680 (64.82) | 332 (48.82) |  |  |  |
| Tumor location | NA |  |  |  |  |  |  |  |  |  |
| Non-cardia |  |  |  |  |  | 590 (56.24) | 250 (53.30) | 0.084 | 0.102 |  |
| Cardia |  |  |  |  |  | 459 (43.76) | 219 (46.70) |  |  |  |
| Abbreviations: AJCC, American Joint Committee on Cancer; NA, not available; TNM, tumor-node-metastasis.  ^a^ Chi-square test for the comparison of mortality between groups.  ^b^ Chi-square test for the comparison of characteristics between the Shanghai cohort and the Jiangsu cohort for each clinical variable. | | | | | | | | | | |

| **Supplementary Table 2.** List of 151 selected natural killer cell pathway-related genes used in the discovery analysis. | | | | |
| --- | --- | --- | --- | --- |
| **Dataset** | **Name of pathway** | **Selected genes ^a^** | **Number of genes** |  |
| GO | GOBP_NATURAL_KILLER_CELL_ACTIVATION | *AP1G1, AXL, BAG6, BLOC1S3, BLOC1S6, CASP8, CD2, CD244, CLNK, CORO1A, DCAF15, ELF4, EMP2, FCGR3A, FGR, GAS6, HAVCR2, HLA-E, HLA-F, IFNA1, IFNA10, IFNA13, IFNA14, IFNA16, IFNA17, IFNA2, IFNA21, IFNA4, IFNA5, IFNA6, IFNA7, IFNA8, IFNB1, IFNE, IFNK, IFNW1, IL12A, IL12B, IL15, IL15RA, IL18, IL18R1, IL2, IL21R, IL23A, IL23R, JAK2, KAT7, KIR3DS1, KLRC1, KLRC2, KLRC3, KLRC4-KLRK1, KLRD1, KLRF2, KLRK1, LAMP1, LEP, MERTK, MICA, MIR130A, NCR1, NCR3, NFIL3, NKG7, PBX1, PGLYRP1, PGLYRP2, PGLYRP3, PIBF1, PIK3CD, PRDM1, PRDX1, PTPN22, PTPRC, RAB27A, RABL3, RASGRP1, RHBDD3, SLAMF1, SLAMF7, SNX27, SP3, STAT5B, TICAM1, TOX, TUSC2, TYK2, TYRO3, TYROBP, ULBP1, ULBP2, ULBP3, UNC13D, VAMP2, VAMP7, ZBTB1, ZNF683* | 98 |  |
| GO | GOBP_NATURAL_KILLER_CELL_MEDIATED_IMMUNITY | *AP1G1, ARL8B, ARRB2, CADM1, CD160, CD2, CD226, CD96, CEACAM1, CEBPG, CLEC12B, CLEC2A, CLNK, CORO1A, CRK, CRTAM, FCGR3A, GFER, GRB2, GZMB, HAVCR2, HCST, HLA-A, HLA-B, HLA-E, HLA-F, HLA-G, IL12A, IL12B, IL18, IL18RAP, IL21, KIF5B, KIR2DL4, KIR3DL1, KLRB1, KLRC1, KLRC2, KLRC3, KLRC4, KLRC4-KLRK1, KLRD1, KLRF2, KLRK1, LAG3, LAMP1, LEP, LGALS9, LILRB1, LYST, MICA, NCR1, NCR3, NECTIN2, NKG7, PIK3R1, PIK3R6, PLEKHM2, PRDX1, PTPN6, PVR, RAB27A, RAET1E, RAET1G, RASGRP1, RASGRP4, RNF19B, SERPINB4, SERPINB9, SH2D1A, SH2D1B, SLAMF6, SLAMF7, STAT5B, TGFB1, TUBB, TUBB4B, ULBP1, ULBP2, ULBP3, UNC13D, VAMP2, VAMP7, VAV1* | 84 |  |
| GO | GOBP_REGULATION_OF_NATURAL_KILLER_CELL_MEDIATED_IMMUNITY | *AP1G1, ARRB2, CADM1, CD160, CD226, CD96, CEACAM1, CLEC12B, CLNK, CRK, CRTAM, GFER, HAVCR2, HLA-A, HLA-B, HLA-E, HLA-F, HLA-G, IL12A, IL12B, IL18RAP, IL21, KIR2DL4, KLRB1, KLRC1, KLRC2, KLRC3, KLRC4, KLRC4-KLRK1, KLRD1, KLRK1, LAG3, LAMP1, LEP, LGALS9, LILRB1, MICA, NCR1, NCR3, NECTIN2, PIK3R6, PVR, RAET1E, RAET1G, RASGRP1, RASGRP4, SERPINB4, SERPINB9, SH2D1A, SH2D1B, SLAMF6, STAT5B, TGFB1, VAV1* | 54 |  |
| GO | GOBP_REGULATION_OF_NATURAL_KILLER_CELL_ACTIVATION | *AXL, BLOC1S3, BLOC1S6, CLNK, DCAF15, FCGR3A, FGR, GAS6, HAVCR2, HLA-E, IL12A, IL12B, IL15, IL15RA, IL18, IL23A, IL23R, JAK2, KLRC1, KLRC2, KLRC3, KLRD1, LEP, MICA, MIR130A, PGL, YRP1, PGLYRP2, PGLYRP3, PIBF1, PRDM1, PTPN22, RASGRP1, RHBDD3, STAT5B, TICAM1, TOX, TYK2, TYROBP, ZBTB1, ZNF683* | 40 |  |
| GO | GOBP_NATURAL_KILLER_CELL_ACTIVATION_INVOLVED_IN_IMMUNE_RESPONSE | *AP1G1, CD244, CORO1A, FCGR3A, HLA-F, IFNA1, IFNA10, IFNA13, IFNA14, IFNA16, IFNA17, IFNA2, IFNA21, IFNA4, IFNA5, IFNA6, IFNA7, IFNA8, IFNB1, IFNE, IFNK, IFNW1, IL12B, KLRC2, KLRF2, LAMP1, NKG7, PGLYRP1, PGL, YRP2, PGLYRP3, RAB27A, UNC13D, VAMP2, VAMP7, ZNF683* | 35 |  |
| GO | GOBP_NATURAL_KILLER_CELL_DIFFERENTIATION | *AXL, GAS6, IL15, IL15RA, KAT7, MERTK, NFIL3, PBX1, PGLYRP1, PGLYRP2, PGLYRP3, PIK3CD, PRDM1, PTPRC, RABL3, RASGRP1, SLAMF1, SP3, STAT5B, TOX, TUSC2, TYRO3, ZBTB1, ZNF683* | 24 |  |
| GO | GOBP_NATURAL_KILLER_CELL_PROLIFERATION | *ELF4, EMP2, FCGR3A, HLA-E, IL12B, IL15, IL18, IL23A, IL23R, JAK2, LEP, PTPN22, SLAMF1, STAT5B, TYK2* | 15 |  |
| GO | GOBP_REGULATION_OF_NATURAL_KILLER_CELL_DIFFERENTIATION | *AXL,GAS6, IL15, IL15RA, PGLYRP1, PGLYRP2, PGLYRP3, PRDM1, RASGRP1, STAT5B, TOX, ZBTB1, ZNF683* | 13 |  |
| GO | GOBP_NATURAL_KILLER_CELL_DEGRANULATION | *AP1G1, CORO1A, FCGR3A, HLA-F, KLRC2, KLRF2, LAMP1, NKG7, RAB27A, UNC13D, VAMP2, VAMP7* | 12 |  |
| GO | GOBP_REGULATION_OF_NATURAL_KILLER_CELL_PROLIFERATION | *FCGR3A, HLA-E, IL12B, IL15, IL18, IL23A, IL23R, JAK2, LEP, PTPN22, STAT5B, TYK2* | 12 |  |
| GO | GOBP_NATURAL_KILLER_CELL_MEDIATED_IMMUNE_RESPONSE_TO_TUMOR_CELL | *CD160, CD226, CEACAM1, CRTAM, HAVCR2, IL12A, IL12B, NECTIN2, NKG7, PVR, TGFB1* | 11 |  |
| GO | GOBP_NATURAL_KILLER_CELL_CHEMOTAXIS | *CCL2, CCL3, CCL4, CCL5, CCL7, KLRC4-KLRK1, KLRK1, PIK3CD, PIK3CG, XCL1* | 10 |  |
| GO | GOBP_REGULATION_OF_NATURAL_KILLER_CELL_MEDIATED_IMMUNE_RESPONSE_TO_TUMOR_CELL | *CD160, CD226, CEACAM1, CRTAM, HAVCR2, IL12A, IL12B, NECTIN2, PVR, TGFB1* | 10 |  |
| G0 | GOBP_NATURAL_KILLER_CELL_CYTOKINE_PRODUCTION | *CD160, CD226, CD96, CLNK, HLA-E, HLA-F, HLA-G, KIR2DL4, RAET1G* | 9 |  |
| GO | GOBP_REGULATION_OF_NATURAL_KILLER_CELL_CHEMOTAXIS | *CCL2, CCL3, CCL4, CCL5, CCL7, KLRC4-KLRK1, KLRK1, XCL1* | 8 |  |
| Total |  |  | **151 ^b^** |  |
| Abbreviations: GO, Gene Ontology; GOBP, Gene Ontology Biological Process.  ^a^ Genes were selected based on the Molecular Signature Database (https://www.gsea-msigdb.org/gsea/msigdb/human/search.jsp).  ^b^ 281 duplicated genes and three genes on the X chromosome had been removed.  Keyword: “natural killer cell”.  Collection: H:hallmark gene sets, CP:canonical pathways, GO:Gene Ontology, and C7:immunologic gene sets.  Organism: Homo sapiens. | | | | |

| **Supplementary Table 3.** Associations of the top 10 principal components and OS of GC in the Shanghai GWAS dataset. | | | | |
| --- | --- | --- | --- | --- |
| **PC** ^a^ | **Beta** | **Standard Error** | **Z** | ***P*** |
| PC1 | 1.516 | 2.686 | 0.564 | 0.573 |
| PC2 | -4.355 | 3.847 | -1.132 | 0.258 |
| PC3 | 2.482 | 3.476 | 0.714 | 0.475 |
| PC4 | -3.265 | 3.478 | -0.939 | 0.348 |
| PC5 | 0.046 | 3.541 | 0.013 | 0.990 |
| PC6 | -2.847 | 3.448 | -0.826 | 0.409 |
| **PC7** | **-7.882** | **3.487** | **-2.260** | **0.024** |
| **PC8** | **-6.644** | **3.463** | **-1.919** | **0.055** |
| PC9 | 0.157 | 3.377 | 0.046 | 0.963 |
| PC10 | 4.407 | 3.416 | 1.290 | 0.197 |
| Abbreviations: GC, gastric cancer; GWAS, genome-wide association study; OS, overall survival; PC, Principal Component.  ^a^ The PC7 and PC8 were used for adjustment for population stratification in the multivariate analysis. | | | | |

| **Supplementary Table 4.** Stratified analysis for associations between unfavorable genotypes and GC survival in the Shanghai GWAS dataset. | | | | | | | | | | | | | |
| --- | --- | --- | --- | --- | --- | --- | --- | --- | --- | --- | --- | --- | --- |
| **Characteristics** | **0-1**  **unfavorable genotype ^a^** | | |  | **2-3**  **unfavorable genotype ^a^** | | |  | **Multivariate**  **analysis ^b^ for OS** | |  | **Log-rank *P*** | ***P* _inter_ ^c^** |
|  | **Frequency** | **Deaths (%)** | **RMST ^d^** |  | **Frequency** | **Deaths (%)** | **RMST ^d^** |  | **HR (95% CI)** | ***P*** |  |  |  |
| Age at diagnosis |  |  |  |  |  |  |  |  |  |  |  |  | 0.213 |
| < 60 | 173 | 40 (23.12) | 53.2/100.2 |  | 855 | 204 (23.86) | 51.9/97.7 |  | 1.38 (0.97-1.96) | 0.074 |  | 0.743 |  |
| ≥ 60 | 209 | 66 (31.58) | 51.6/93.7 |  | 974 | 434 (44.56) | 47.1/82.1 |  | 1.78 (1.37-2.31) | <0.001 |  | <0.001 |  |
| Sex |  |  |  |  |  |  |  |  |  |  |  |  | 0.583 |
| Female | 104 | 26 (25.00) | 53.2/99.0 |  | 542 | 186 (34.32) | 49.3/89.6 |  | 1.67 (1.10-2.53) | 0.016 |  | 0.050 |  |
| Male | 278 | 80 (28.78) | 52.0/95.8 |  | 1,287 | 452 (35.12) | 49.4/89.4 |  | 1.53 (1.20-1.94) | <0.001 |  | 0.032 |  |
| Smoking status |  |  |  |  |  |  |  |  |  |  |  |  | 0.703 |
| Never | 238 | 66 (27.73) | 52.5/96.9 |  | 1,186 | 402 (33.90) | 49.7/90.3 |  | 1.53 (1.18-1.99) | 0.001 |  | 0.045 |  |
| Current | 27 | 14 (51.85) | 41.8/69.5 |  | 103 | 71 (68.93) | 33.5/49.0 |  | 2.21 (1.16-4.22) | 0.016 |  | 0.061 |  |
| Former | 117 | 26 (22.22) | 54.4/102.2 |  | 540 | 165 (30.56) | 51.6/94.8 |  | 1.37 (0.90-2.09) | 0.140 |  | 0.068 |  |
| Drinking status |  |  |  |  |  |  |  |  |  |  |  |  | 0.681 |
| No | 267 | 77 (28.84) | 52.2/95.8 |  | 1,369 | 479 (34.99) | 49.3/89.2 |  | 1.61 (1.26-2.05) | <0.001 |  | 0.030 |  |
| Yes | 115 | 29 (25.22) | 52.7/98.7 |  | 460 | 159 (34.57) | 49.7/90.1 |  | 1.43 (0.96-2.13) | 0.081 |  | 0.061 |  |
| TNM stage |  |  |  |  |  |  |  |  |  |  |  |  | **0.047** |
| I | 96 | 7 (7.29) | 57.8/113.7 |  | 550 | 34 (6.18) | 59.2/116.2 |  | 0.89 (0.38-2.06) | 0.785 |  | 0.715 |  |
| II | 112 | 21 (18.75) | 57.1/107.6 |  | 452 | 99 (21.90) | 54.6/102.6 |  | 1.20 (0.74-1.92) | 0.463 |  | 0.374 |  |
| III | 174 | 78 (44.83) | 46.2/80.3 |  | 827 | 505 (61.06) | 40.0/64.6 |  | 1.71 (1.35-2.18) | <0.001 |  | <0.001 |  |
| Chemotherapy |  |  |  |  |  |  |  |  |  |  |  |  | 0.903 |
| No | 127 | 28 (22.05) | 52.0/98.8 |  | 691 | 172 (24.89) | 52.3/98.5 |  | 1.54 (1.02-2.31) | 0.040 |  | 0.496 |  |
| Yes | 255 | 78 (30.59) | 52.5/95.6 |  | 1,138 | 466 (40.95) | 47.6/84.0 |  | 1.59 (1.25-2.02) | <0.001 |  | 0.001 |  |
| Radiotherapy |  |  |  |  |  |  |  |  |  |  |  |  | 0.445 |
| No | 364 | 100 (27.47) | 52.3/96.7 |  | 1,745 | 593 (33.98) | 49.7/90.2 |  | 1.54 (1.25-1.91) | <0.001 |  | 0.011 |  |
| Yes | 18 | 6 (33.33) | 52.9/96.2 |  | 84 | 45 (53.57) | 42.8/73.9 |  | 2.01 (0.82-4.92) | 0.126 |  | 0.092 |  |
| Abbreviations: CI, confidence interval; GC, gastric cancer; GWAS, genome-wide association study; HR, hazard ratio; OS, overall survival; RMST, restricted mean survival time; TNM, tumor-node-metastasis.  ^a^ Unfavorable genotypes were *CD160* rs9728526 AG+GG, *MERTK* rs114788905 GG+GA, and *IL15* rs140007893 TT.  ^b^ Adjusted for age at diagnosis, sex, smoking status, drinking status, TNM stage, chemotherapy, radiotherapy, PC7, and PC8.  ^c^ *P* _inter_: *P* value for interaction analysis between characteristics and unfavorable genotypes.  ^d^ The RMST was measured over a 5-year/10-year period. | | | | | | | | | | | | | |

| **Supplementary Table 5.** The NRIs and IDIs of the clinical model combining SNPs for survival prediction in gastric cancer patients. | | | |
| --- | --- | --- | --- |
| **Time points** | **NRI (95% CI)** | **IDI (95%CI)** | ***P* for IDI** |
| Five year | 0.014 (-0.008-0.037) | 0.006 (0.001-0.014) | 0.010 |
| Ten year | 0.033 (0.010-0.053) | 0.007 (0.001-0.015) | 0.016 |
| Abbreviations: NRI, net reclassification improvement; IDI, integrated discrimination improvement; CI: confidence interval. | | | |

| **Supplementary Table 6.** Function prediction for the three significant SNPs in both cohorts. | | | | | | | | | | | | |
| --- | --- | --- | --- | --- | --- | --- | --- | --- | --- | --- | --- | --- |
| **SNP** | **Gene** | **Chr** | **Genotyped** | **RegulomeDB**  **2.2 ^a^** | **3DSNPv2.0 ^b^**  **score** | **Haploreg v4.2 ^c^** | | | | | **SNPinfo**  **Web Server ^d^** | **Ensembl**  **Variant**  **Effect**  **Predictor ^e^** |
|  |  |  |  |  |  | **Promoter histone marks** | **Enhancer histone**  **marks** | **DNAse** | **Motifs changed** | **Selected eQTL hits** |  |  |
| rs9728526 | *CD160* | 1 | No | 5 | 1.13 | -- | ESDR, BLD | -- | 13 altered motifs | 9 hits | TFBS | upstream_gene_variant |
| rs114788905 | *MERTK* | 2 | No | 3a | 2.00 | -- | -- | -- | 4 altered motifs | -- | -- | intron_variant |
| rs140007893 | *IL15* | 4 | No | 6 | 1.43 | -- | -- | -- | 4 altered motifs | -- | -- | intron_variant |
| Abbreviations: Chr, chromosome; eQTL, expression quantitative trait loci; SNP, single nucleotide polymorphism; TFBS, transcription factor binding site.  ^a^ RegulomeDB 2.2: http://regulomedb.org/.  ^b^ 3DSNP v2.0: https://omic.tech/3dsnpv2/.  ^c^ HaploReg v4.2: https://pubs.broadinstitute.org/mammals/haploreg/haploreg.php.  ^d^ SNPinfo Web Server: https://snpinfo.niehs.nih.gov/snpinfo/snpfunc.html.  ^e^ Ensembl Variant Effect Predictor: https://grch37.ensembl.org/Tools/VEP. | | | | | | | | | | | | |

| **Supplementary Table 7.** Available and non-significant eQTL results for independent SNPs across diverse gastric cell type from the scGaTE website (N = 203). | | | | | | | |
| --- | --- | --- | --- | --- | --- | --- | --- |
| **Gastric cell type** | **SNP** | **Position**  **(GRCh37)** | **Reference**  **allele** | **Effect**  **allele** | **Gene ID** | **Gene**  **symbol** | ***P*-value** |
| Artery-derived endothelial cells | rs114788905 | 2:112757584 | A | G | ENSG00000153208 | *MERTK* | 0.915 |
| CD4^+^ T cells | rs114788905 | 2:112757584 | A | G | ENSG00000153208 | *MERTK* | 0.545 |
| Chief cells | rs114788905 | 2:112757584 | A | G | ENSG00000153208 | *MERTK* | 0.639 |
| Endocrine cells | rs114788905 | 2:112757584 | A | G | ENSG00000153208 | *MERTK* | 0.633 |
| Intestinal-type cells | rs114788905 | 2:112757584 | A | G | ENSG00000153208 | *MERTK* | 0.166 |
| M1-like macrophages | rs114788905 | 2:112757584 | A | G | ENSG00000153208 | *MERTK* | 0.772 |
| M2-like macrophages | rs114788905 | 2:112757584 | A | G | ENSG00000153208 | *MERTK* | 0.586 |
| Mucous neck cells | rs114788905 | 2:112757584 | A | G | ENSG00000153208 | *MERTK* | 0.704 |
| Mucous pit cells | rs114788905 | 2:112757584 | A | G | ENSG00000153208 | *MERTK* | 0.876 |
| Myofibroblasts | rs114788905 | 2:112757584 | A | G | ENSG00000153208 | *MERTK* | 0.266 |
| Plasma cells | rs114788905 | 2:112757584 | A | G | ENSG00000153208 | *MERTK* | 0.076 |
| Stalk-like endothelial cells | rs114788905 | 2:112757584 | A | G | ENSG00000153208 | *MERTK* | 0.770 |
| Tip-like endothelial cells | rs114788905 | 2:112757584 | A | G | ENSG00000153208 | *MERTK* | 0.592 |
| Plasma cells | rs140007893 | 4:142586291 | T | A | ENSG00000164136 | *IL15* | 0.556 |
| Abbreviations: eQTL, expression quantitative trait loci; SNP, single nucleotide polymorphism. | | | | | | | |

| **Supplementary Table 8.** Available and significant eQTL results for three independent SNPs across diverse immune cell types from three online sc-eQTL databases. | | | | | | | | | | | |
| --- | --- | --- | --- | --- | --- | --- | --- | --- | --- | --- | --- |
| **Database** | **Immune cell type** | **Gene**  **symbol** | **Gene ID** | **SNP** | **Position**  **(GRCh37)** | **Ref**  **allele** | **Effect**  **allele** | ***P*-value** | **Effect**  **size** | **Standard error** | **Sample size** |
| FIVEx platform ^a^ | Memory Th1 cells | *MERTK* | ENSG00000153208 | rs114788905 | 2:112757584 | A | G | 0.009 | 0.48 | 0.18 | 82 |
|  | Memory Th2 cells | *MERTK* | ENSG00000153208 | rs114788905 | 2:112757584 | A | G | 0.043 | 0.39 | 0.19 | 89 |
|  | Naïve CD4^+^ T cells | *MERTK* | ENSG00000153208 | rs114788905 | 2:112757584 | A | G | 0.003 | 0.72 | 0.24 | 88 |
|  | Naïve monocytes | *MERTK* | ENSG00000153208 | rs114788905 | 2:112757584 | A | G | 0.013 | -0.53 | 0.21 | 200 |
|  | Naïve Treg cells | *MERTK* | ENSG00000153208 | rs114788905 | 2:112757584 | A | G | 0.008 | 0.67 | 0.25 | 89 |
|  | Memory Treg cells | *IL15* | ENSG00000164136 | rs140007893 | 4:142586291 | T | A | 0.017 | -1.22 | 0.50 | 89 |
| scQTLbase ^b^ | CD8^+^ T cells | *CD160* | ENSG00000117281 | rs9728526 | 1:145716763 | G | A | 1.1e-10 | -0.35 | 0.05 | -- |
|  | Memory CD4^+^ T cells | *CD160* | ENSG00000117281 | rs9728526 | 1:145716763 | G | A | 4.0e-5 | -0.24 | 0.06 | -- |
|  | Memory T cells | *CD160* | ENSG00000117281 | rs9728526 | 1:145716763 | G | A | 2.8e-8 | -0.30 | 0.05 | -- |
| Single-cell eQTL Hub ^c^ | Naïve CD8^+^ T cells | *CD160* | ENSG00000117281 | rs9728526 | 1:145716763 | G | A | 0.012 | -- | -- | -- |
|  | Naïve Treg cells | *MERTK* | ENSG00000153208 | rs114788905 | 2:112757584 | A | G | 0.023 | 0.48 | -- | -- |
|  | Naïve CD4^+^ T cells | *MERTK* | ENSG00000153208 | rs114788905 | 2:112757584 | A | G | 0.028 | 0.46 | -- | -- |
|  | CD16^+^ monocytes | *IL15* | ENSG00000164136 | rs140007893 | 4:142586291 | T | A | 0.012 | -0.30 | -- | -- |
|  | Classical monocytes | *IL15* | ENSG00000164136 | rs140007893 | 4:142586291 | T | A | 0.006 | -0.30 | -- | -- |
|  | Intermediate monocytes | *IL15* | ENSG00000164136 | rs140007893 | 4:142586291 | T | A | 0.001 | -0.45 | -- | -- |
|  | Non-classical monocytes | *IL15* | ENSG00000164136 | rs140007893 | 4:142586291 | T | A | 0.010 | -0.37 | -- | -- |
| Abbreviations: Ref, reference; sc-eQTL, single-cell expression quantitative trait loci; SNP, single nucleotide polymorphism; Treg cell, regulatory T cell.  ^a^ FIVEx platform: https://fivex.sph.umich.edu/.  ^b^ scQTLbase: http://bioinfo.szbl.ac.cn/scQTLbase/Home/.  ^c^ Single-cell eQTL Hub: https://www.sqraolab.com/scqtl/. | | | | | | | | | | | |

| **Supplementary Table 9.** Available cis-sQTL results for the three independent SNPs across blood, stomach, and various immune cell types from the FIVEx platform. | | | | | | | | | | | |
| --- | --- | --- | --- | --- | --- | --- | --- | --- | --- | --- | --- |
| **Name** | **Transcript ID ^a^** | **Blood/immune cells** | **SNP** | **Position**  **(GRCh37)** | **Ref**  **allele** | **Effect**  **allele** | **Gene symbol** | ***P*-value** | **Effect size** | **SE** | **Sample**  **size** |
| CD160-006 | ENST00000369288 | Blood | rs9728526 | 1:145716763 | G | A | *CD160* | 0.003 | -0.13 | 0.04 | 379 |
|  |  | Memory Th1 cells | rs9728526 | 1:145716763 | G | A | *CD160* | 0.030 | -0.42 | 0.19 | 82 |
|  |  | Memory Th1-17 cells | rs9728526 | 1:145716763 | G | A | *CD160* | 0.043 | 0.32 | 0.16 | 88 |
|  |  | Memory Tfh cells | rs9728526 | 1:145716763 | G | A | *CD160* | 0.002 | 0.57 | 0.18 | 89 |
|  |  | Naïve B cells | rs9728526 | 1:145716763 | G | A | *CD160* | 0.010 | 0.39 | 0.15 | 91 |
|  |  | Naïve CD4^+^ T cells | rs9728526 | 1:145716763 | G | A | *CD160* | 0.020 | 0.32 | 0.14 | 88 |
|  |  | Naïve natural kill cells ^b^ | rs9728526 | 1:145716763 | G | A | *CD160* | 0.005 | -0.09 | 0.03 | 90 |
|  |  | Naïve natural kill cells ^b^ | rs9728526 | 1:145716763 | G | A | *CD160* | 0.017 | -0.08 | 0.03 | 90 |
|  |  | Stomach | rs9728526 | 1:145716763 | G | A | *CD160* | 0.038 | -0.24 | 0.11 | 188 |
| CD160-201 | ENST00000235933 | Blood ^c^ | rs9728526 | 1:145716763 | G | A | *CD160* | 0.010 | -0.18 | 0.07 | 379 |
|  |  | Blood ^c^ | rs9728526 | 1:145716763 | G | A | *CD160* | 0.017 | -0.16 | 0.07 | 471 |
|  |  | Memory Th1 cells | rs9728526 | 1:145716763 | G | A | *CD160* | 0.002 | 0.54 | 0.17 | 82 |
|  |  | Memory Treg cells | rs9728526 | 1:145716763 | G | A | *CD160* | 0.032 | 0.37 | 0.17 | 89 |
|  |  | Naïve CD4^+^ T cells | rs9728526 | 1:145716763 | G | A | *CD160* | 0.032 | 0.35 | 0.16 | 88 |
|  |  | T cells | rs9728526 | 1:145716763 | G | A | *CD160* | 0.049 | 0.22 | 0.11 | 184 |
| CD160-202 | ENST00000401557 | Blood | rs9728526 | 1:145716763 | G | A | *CD160* | 1.8e-4 | 0.24 | 0.07 | 471 |
|  |  | Memory Tfh cells | rs9728526 | 1:145716763 | G | A | *CD160* | 0.040 | -0.35 | 0.17 | 89 |
|  |  | Naïve CD16^+^ monocytes | rs9728526 | 1:145716763 | G | A | *CD160* | 0.039 | -0.42 | 0.20 | 90 |
| MERTK-001 | ENST00000295408 | Memory Th2 cells ^b^ | rs114788905 | 2:112757584 | A | G | *MERTK* | 0.005 | -0.62 | 0.21 | 89 |
|  |  | Memory Th2 cells ^b^ | rs114788905 | 2:112757584 | A | G | *MERTK* | 0.028 | -0.42 | 0.19 | 89 |
|  |  | Monocytes ^b^ | rs114788905 | 2:112757584 | A | G | *MERTK* | 0.008 | -0.28 | 0.11 | 191 |
|  |  | Monocytes ^b^ | rs114788905 | 2:112757584 | A | G | *MERTK* | 0.043 | -0.28 | 0.13 | 191 |
|  |  | Naïve macrophages ^b^ | rs114788905 | 2:112757584 | A | G | *MERTK* | 0.011 | -0.70 | 0.27 | 163 |
|  |  | Naïve macrophages ^b^ | rs114788905 | 2:112757584 | A | G | *MERTK* | 0.026 | -0.17 | 0.08 | 163 |
|  |  | T cells | rs114788905 | 2:112757584 | A | G | *MERTK* | 0.038 | 0.52 | 0.25 | 184 |
| MERTK-002 | ENST00000409780 | Blood | rs114788905 | 2:112757584 | A | G | *MERTK* | 0.018 | 0.27 | 0.11 | 471 |
| MERTK-005 | ENST00000449344 | Memory Tfh cells | rs114788905 | 2:112757584 | A | G | *MERTK* | 0.014 | 0.53 | 0.21 | 89 |
|  |  | Memory Treg cells ^b^ | rs114788905 | 2:112757584 | A | G | *MERTK* | 0.017 | -0.55 | 0.22 | 89 |
|  |  | Memory Treg cells ^b^ | rs114788905 | 2:112757584 | A | G | *MERTK* | 0.028 | -0.49 | 0.22 | 89 |
|  |  | Naïve macrophages | rs114788905 | 2:112757584 | A | G | *MERTK* | 0.040 | -0.22 | 0.11 | 163 |
| MERTK-201 | ENST00000421804 | Memory Treg cells | rs114788905 | 2:112757584 | A | G | *MERTK* | 0.012 | 0.55 | 0.21 | 89 |
|  |  | Naïve CD16^+^ monocytes | rs114788905 | 2:112757584 | A | G | *MERTK* | 0.013 | 0.55 | 0.22 | 90 |
|  |  | Naïve CD4^+^ T cells | rs114788905 | 2:112757584 | A | G | *MERTK* | 0.028 | 0.46 | 0.21 | 88 |
|  |  | Naïve macrophages | rs114788905 | 2:112757584 | A | G | *MERTK* | 0.021 | 0.17 | 0.07 | 163 |
|  |  | Naïve monocytes | rs114788905 | 2:112757584 | A | G | *MERTK* | 0.043 | 0.78 | 0.38 | 200 |
|  |  | Naïve natural kill cells | rs114788905 | 2:112757584 | A | G | *MERTK* | 0.025 | 0.53 | 0.23 | 90 |
|  |  | T cells | rs114788905 | 2:112757584 | A | G | *MERTK* | 0.025 | 0.61 | 0.27 | 184 |
| IL15-001 | ENST00000296545 | Memory Th1-17 cells | rs140007893 | 4:142586291 | T | A | *IL15* | 0.035 | -1.20 | 0.56 | 88 |
|  |  | Naïve CD16^+^ monocytes | rs140007893 | 4:142586291 | T | A | *IL15* | 0.047 | 0.70 | 0.34 | 90 |
|  |  | Naïve Treg cells | rs140007893 | 4:142586291 | T | A | *IL15* | 0.022 | 1.52 | 0.65 | 89 |
| IL15-002 | ENST00000320650 | Naïve CD8^+^ T cells | rs140007893 | 4:142586291 | T | A | *IL15* | 0.015 | -1.19 | 0.48 | 89 |
| IL15-003 | ENST00000477265 | Memory Tfh cells | rs140007893 | 4:142586291 | T | A | *IL15* | 0.011 | -0.83 | 0.32 | 89 |
|  |  | Memory Th17 cells | rs140007893 | 4:142586291 | T | A | *IL15* | 0.012 | -0.92 | 0.36 | 89 |
|  |  | Naïve Treg cells | rs140007893 | 4:142586291 | T | A | *IL15* | 0.001 | -2.09 | 0.61 | 89 |
| IL15-004 | ENST00000514653 | Memory Th1-17 cells | rs140007893 | 4:142586291 | T | A | *IL15* | 0.016 | 1.88 | 0.76 | 88 |
|  |  | Memory Th2 cells | rs140007893 | 4:142586291 | T | A | *IL15* | 0.043 | -1.69 | 0.82 | 89 |
|  |  | Memory Th17 cells ^b^ | rs140007893 | 4:142586291 | T | A | *IL15* | 0.012 | 0.93 | 0.36 | 89 |
|  |  | Memory Th17 cells ^b^ | rs140007893 | 4:142586291 | T | A | *IL15* | 0.018 | 1.08 | 0.45 | 89 |
|  |  | Naïve Treg cells | rs140007893 | 4:142586291 | T | A | *IL15* | 0.048 | -1.37 | 0.68 | 89 |
| IL15-008 | ENST00000529613 | Memory Th1-17 cells | rs140007893 | 4:142586291 | T | A | *IL15* | 0.001 | -2.62 | 0.77 | 88 |
| Abbreviations: Ref, Reference; SE, Standard error; SNP, single nucleotide polymorphism; sQTL, splicing quantitative trait loci; Tfh cells, follicular T helper cells; Treg cells, regulatory T cells.  ^a^ Protein-coding transcript (obtained from the Ensembl database: https://grch37.ensembl.org/index.html).  ^b^ Different splicing sites.  ^c^ Different studies. | | | | | | | | | | | |
