# Supplementary material for: Genetic variants of CD160, MERTK, and IL15 in natural killer cell-related pathway predict gastric cancer survival
Source: Front Immunol. 2026 Apr 15;17:1765825. doi: 10.3389/fimmu.2026.1765825 (PMC13125121; doi:10.3389/fimmu.2026.1765825)
Supplement: Supplementary file 2 [file DataSheet2.docx]

**Supplementary Figure 1.** Manhattan plot in the Shanghai GWAS dataset and the Jiangsu GWAS dataset. (A) Manhattan plot for 12,476 SNPs of natural killer cell-related genes in the Shanghai GWAS dataset; (B) Manhattan plot for 165 SNPs of natural killer cell-related genes in the Jiangsu GWAS dataset. The blue horizontal line indicates *P* = 0.05 and the red line indicates BFDP = 0.80.

Abbreviations: BFDP, Bayesian False Discovery Probability; GWAS, genome-wide association study; SNP, single nucleotide polymorphism.

**Supplementary Figure 2.** Regional association plots for the two independent SNPs in the natural killer cell-related genes.

Regional association plots included 100kb up or downstream of (A) *CD160*, (B) *MERTK*, and (C) *IL15*. Data points are colored according to the level of LD of each pair of SNPs based on the hg19/1000 Genomes Asian population. The left-hand y-axis shows the association *P*-value of individual SNPs in the Shanghai cohort, which is plotted as -log10 (*P*) against chromosomal base-pair position. The right-hand y-axis shows the recombination rate estimated from HapMap Data Rel 22/phase II Asian population.

Abbreviations: chr, chromosome; LD, linkage disequilibrium; SNP, single nucleotide polymorphism.

**Supplementary Figure 3**. Prediction of OS with genotypes of *CD160* rs9728526*, MERTK* rs114788905, and *IL15* rs140007893 in the Shanghai GWAS dataset. KM survival curves of *CD160* rs9728526 for OS with (A) additive model, (B) dominant model, and (C) recessive model; *MERTK* rs114788905 for OS with (D) additive model, (E) dominant model, and (F) recessive model; *IL15* rs140007893 for OS with (G) additive model, (H) dominant model, and (I) recessive model.

Abbreviations: GWAS, genome-wide association study; KM, Kaplan–Meier; OS, overall survival.

**Supplementary Figure 4.** Time-dependent ROC curves and AUCs were utilized to evaluate the prognostic ability of the three independent SNPs (rs9728526, rs114788905, and rs140007893) and other independent prognostic factors for overall survival in the Shanghai GC cohort at (A) 5 and (B) 10 years.

Abbreviations: AUC, area under the curve; GC, gastric cancer; ROC, receiver operating characteristic; SNP, single nucleotide polymorphism; TNM, tumor-node-metastasis.

**Supplementary Figure 5.** GC survival prediction with three independent SNPs (i.e., rs9728526 A>G, rs114788905 G>A, and rs140007893 T>A) by AUC and ROC curve at the 12^th^, 36^th^, 60^th^, 96^th^, and 120^th^ month. (A) Time-dependent AUC analysis of OS based on age at diagnosis, sex, smoking status, drinking status, TNM stage, chemotherapy, radiotherapy, PC7, PC8, and the three SNPs. The ROC curve evaluation for (B) OS at 12^th^ month, (C) OS at 36^th^ month, (D) OS at 60^th^ month, (E) OS at 96^th^ month, and (F) OS at 120^th^ month.

Abbreviations: AUC, area under the curve; GC, gastric cancer; OS, overall survival; PC, Principal Component; ROC, receiver operating characteristic; SNP, single nucleotide polymorphism.

**Supplementary Figure 6.** Functional annotation for the three independent SNPs. (A) Location and regulatory features of rs9728526 using data from the ENCODE project annotated with the UCSC genome browser; (B) ChIP-sequencing data from the HaploReg v4.2 database suggested that rs9728526 is involved in promoter function in stomach tissues; (C) location and regulatory features of rs114788905 using data from the ENCODE project annotated with the UCSC genome browser; (D) ChIP-sequencing data from the HaploReg v4.2 database indicated rs114788905 is involved in promoter function in stomach tissues; (E) location and regulatory features of rs140007893 using data from the ENCODE project annotated with the UCSC genome browser.

Abbreviations: SNP, single nucleotide polymorphism.

**Supplementary Figure 7.** Analyses of the effects of the three independent SNPs on transcription factor binding using the JASPAR database and correlation analysis.

The rs9728526 may influence the DNA-binding motifs of transcription factors (A) FEZF2, (B) KLF2, (C) TCF3, and (D) TCF12; (E) the rs114788905 may affect a DNA-binding motif of the transcription factor ZXDB; (F) the rs140007893 may impact a DNA-binding motif of the transcription factor HMBOX1; (G) scatter plots showed correlations between *CD160* expression and the expression of *FEZF2*, *KLF2*, *TCF3*, and *TCF12* in the TCGA dataset comprising 408 stomach adenocarcinoma tissues; (H) TCGA data indicate a positive correlation in mRNA expression levels between *MERTK* and *ZXDB* in stomach adenocarcinoma; (I) TCGA data reveal a positive correlation between *IL15* and *HMBOX1* mRNA expression levels in stomach adenocarcinoma.

Abbreviations: SNP, single nucleotide polymorphism; TCGA, the Cancer Genome Atlas.

**Supplementary Figure 8.** The results of eQTL analyses.

The *MERTK* rs114788905 A allele was not associated with mRNA expression levels of *MERTK* in (A) whole blood samples, (B) normal stomach tissues, and (C) normal esophageal mucosa tissues from the GTEx project; (D) both the *CD160* rs9728526 G allele and the *MERTK* rs114788905 A allele were significantly associated with higher mRNA expression levels of *CD160* and *MERTK* in human blood samples from the eQTLGen database, respectively.

Abbreviations: Chr, chromosome; eQTL, expression quantitative trait loci; FDR, false discovery rate; GTEx, Genotype-Tissue Expression; Pos, position; SNP, single nucleotide polymorphism.


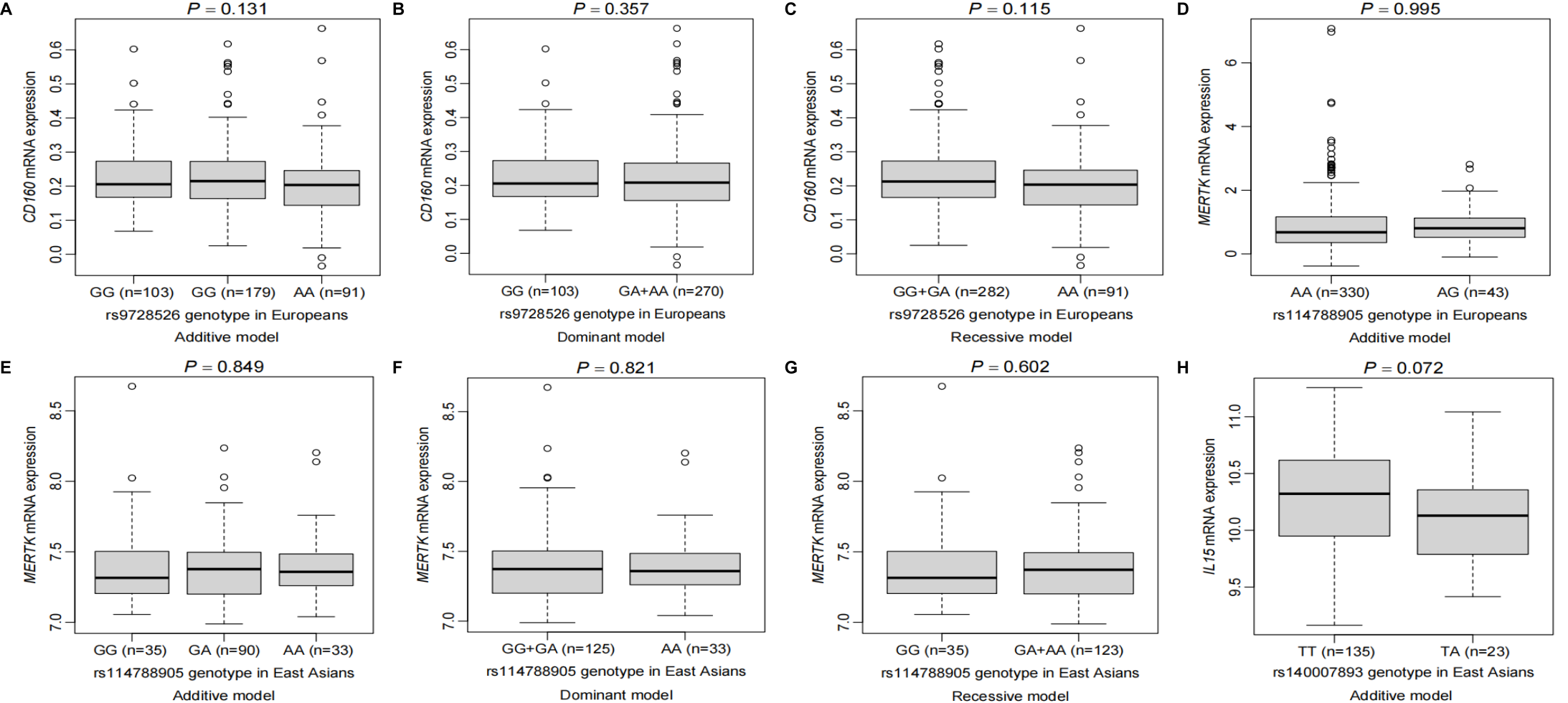


**Supplementary Figure 9.** The results of eQTL analyses for the three independent SNPs in lymphoblastoid cell lines from the 1000 Genomes Project.

The *CD160* rs9728526 G allele was not associated with mRNA expression levels of *CD160* in European individuals across the (A) additive, (B) dominant, and (C) recessive models; (D) the *MERTK* rs114788905 A allele showed no association with *MERTK* mRNA expression levels in European individuals under the additive model; the *CD160* rs9728526 G allele was not found to be associated with *MERTK* mRNA expression levels in East Asian individuals across the (E) additive, (F) dominant, and (G) recessive models; (H) the *IL15* rs140007893 A allele showed no association with *IL15* mRNA expression levels in East Asian individuals under the additive model.

Abbreviations: eQTL, expression quantitative trait loci; SNP, single nucleotide polymorphism.

**Supplementary Figure 10.** The results of QTL analyses.

Heatmaps showing the results of (A) mQTL and (B) bQTL for *CD160* rs9728526 from the QTLbase database; heatmaps showing the results of (C) mQTL and (D) hQTL for *MERTK* rs114788905 from the QTLbase database.

Abbreviations: bQTL, transcription factor binding quantitative trait loci; hQTL, histone modification quantitative trait loci; mQTL, methylation quantitative trait loci; QTL, quantitative trait loci.

**Supplementary Figure 11**. The correlation between the mRNA expression of *CD160*, *MERTK*, and *IL15* and TNM stage in GC.

(A) the violin plots showing the relationship between the expression of *CD160*, *MERTK*, and *IL15* and the TNM stage; (B) the violin plots showing differences in the expression levels of *CD160*, *MERTK*, and *IL15* between TNM stage I-II and III-IV groups; (F) the violin plots showing no differences in the expression levels of *CD160*, *MERTK*, and *IL15* between TNM stage I-III and IV groups;

Abbreviations: GC, gastric cancer; TCGA, the Cancer Genome Atlas; TNM, tumor-node-metastasis.

**Supplementary Figure 12.** The results of the GO enrichment analyses and GSEA from the CAMOIP database.

The top 10 significantly relevant biological processes correlated with high expression of (A) *CD160*, (C) *MERTK,* and (E) *IL15;* the GSEA for significant hallmarks correlated with the elevated expression of (B) *CD160*, (D) *MERTK*, and (F) *IL15* in STAD.

Abbreviations: GO, gene ontology; GOBP, gene ontology biological process; GSEA, gene set enrichment analysis; NES, normalized enrichment score; STAD, stomach adenocarcinoma.

**Supplementary Figure 13**. The results of immune infiltration analyses.

The scatter plots showed a positive correlation between stromal, immune and ESTIMATE scores and the expression of (A) *CD160*, (B) *MERTK*, and (C) *IL15* in STAD, using the Spearman correlation test; the box plots compared scores for MHC molecules, effector cells, suppressor cells, and checkpoint molecules between the low- and high-expression groups of (D) *CD160*, (E) *MERTK*, and (F) *IL15*; (G) the scatter plot showed that the expression of *CD160* was not correlated with the infiltration levels of NK cells in STAD; the scatter plots revealed that the expression of (H) *MERTK* and (I) *IL15* was positively correlated with the infiltration levels of NK cells; (J) the box plot showed no differences in NK cell enrichment score between the low- and high-expression groups of *CD160* in STAD using the ssGSEA algorithm; (K) the box plots showed that the abundance of fibroblasts was significantly higher in the *IL15* high-expression group compared to the low-expression group, while no significant difference in the abundance of endothelial cells was observed between the low- and high-expression groups of *IL15* in STAD using the MCPcounter algorithm. *** *P* < 0.001, **** *P* < 0.0001.

Abbreviations: MCPcounter, Microenvironment Cell Populations counter; NK cell; natural killer cell; ns, not significant; ssGSEA, single-sample gene set enrichment analysis; STAD, stomach adenocarcinoma; TCGA, the Cancer Genome Atlas.
